# Sex- and stage-dependent expression of gonadal soma-derived factor paralogues reveals functional and evolutionary divergence in European sea bass (*Dicentrarchus labrax*)

**DOI:** 10.3389/fendo.2026.1779674

**Published:** 2026-03-02

**Authors:** Alessia Mascoli, Cinta Zapater, Joan Pizarro-Gomez, Ana Gómez

**Affiliations:** 1 Department of Fish Physiology and Biotechnology, Instituto de Acuicultura Torre de la Sal, Consejo Superior de Investigaciones Científicas (CSIC), Torre la Sal, Castellón, Spain; 2 Escuela Técnica Superior de Ingeniería Agnonómica, Alimentaria y de Biosistemas, Universidad Politécnica de Madrid, Madrid, Spain

**Keywords:** evolution, gametogenesis, gene duplication, gonadal factors, reproduction, teleost

## Abstract

**Introduction:**

The gonadal soma-derived factor (Gsdf) is a member of the Transforming Growth Factor-β (TGF-β) superfamily, with key roles in teleost reproduction, particularly in males. Previously considered teleost-specific, its presence in non-teleostvertebrates indicates a more ancient evolutionary origin. It is still classified as an orphan ligand with uncharacterized signaling pathways, and its evolution, regulatory mechanisms, and functional divergence remain unclear.

**Methods:**

The roesent work provides a comprehensive characterization of two *gsdf* paralogues in European sea bass (*Dicentrarchus labrax*), integrating phylogenetic, synteny, transcriptional regulation, and protein localization analyses, together with stage-specific gene expression profiling during ontogeny, puberty onset and adult reproductive cycle.

**Results:**

Phylogenetic reconstruction of 31 species revealed that *gsdf* duplications are independent, lineage-specific events unrelated to the teleost-specific whole genome duplication (3R). Synteny analyses showed that *gsdf1* retains strong conservation with ancestral loci, whereas *gsdf2* resides in a distinct but conserved genomic context, suggesting complex rearrangement. Comparative promoter analysis identified conserved transcription factor binding sites, supporting a shared regulatory framework across teleosts. Expression profiling revealed that both paralogues are gonad-enriched, expressed from early gonadal differentiation, and downregulated at the onset of precocious male puberty. In adults, *gsdf1* was predominantly expressed in males, especially during pre-meiotic stages, while *gsdf2* was more abundant in females, particularly in follicular cells during pre-vitellogenesis. Immunolocalization confirmed stage- and sex-specific presence in Sertoli and follicular cells, indicating local action.

**Conclusions:**

These results support the subfunctionalization of *gsdf* paralogues in sea bass, with sexspecific partitioning of reproductive roles, and provide new insight into the evolutionary plasticity of gonad-related genes in teleosts.

## 1 Introduction

The gonadal soma-derived factor (Gsdf) is a secretory protein belonging to the Transforming Growth Factor-β (TGF-β) superfamily, an extensive group of extracellular ligands, surface receptors, and signaling modulators (1) heavily involved in numerous physiological processes (2, 3), including reproduction (4–6). Although *gsdf* has recently been identified in non-teleost species through *in silico* analysis (7), this gene is considered primarily restricted to teleosts, given its absence in tetrapods such as anurans, mammals, and reptiles (8). The protein is composed of a precursor domain, which includes a signal peptide and is cleaved upon secretion, and a mature TGF-β domain (7). The conserved C-terminal TGF-β domain, characterized by a cysteine knot structure, represents a common feature of all ligands included in this superfamily and is essential for dimerization and receptor binding. Interestingly, the Gsdf cysteine knot lacks a glycine residue between the second and third cysteines, forming a distinctive signature that sets it apart from many other TGF-β proteins including Amh, Gdf9, and Inha (9–11). In line with this structural distinction of the protein, phylogenetic analyses consistently place *gsdf* genes in a separate clade from other TGF-β ligands, supporting its unique molecular features (8, 11–14). So far, this gonadal factor is considered an orphan ligand, with its specific receptor yet to be identified. However, yeast-two-hybrid assays suggest potential interactions with TGF-β type II receptors, Amhr2 (Anti-Müllerian hormone receptor 2) and Bmpr2 (Bone Morphogenetic Protein Receptor 2), thereby laying the foundation for future investigations into the signaling pathway of this gene (15).

First identified in rainbow trout (*Oncorhynchus mykiss*) as a genital ridge-specific gene (16), *gsdf* subsequently gained interest and thus has been found in numerous other teleost species, emerging as a key component in the cascade of the reproductive regulation in males and across species exhibiting distinct reproductive strategies. In a few species, it has been identified in duplicate, with both paralogues functionally implicated in reproduction and strongly associated with the gonadal activity, as documented in rainbow trout (17), in European sea bass (*Dicentrarchus labrax*) (18), and in gibel carp (*Carassius gibelio*) (19).

Among the TGF-β members acting as master sex-determining gene (5, 20), *gsdf* has been proposed to play such a role in some species, originated by allelic diversification in Philippine medaka (*Oryzias luzonensis*) (21) and sablefish (*Anopoploma fimbria*) (22), or by heterochiasmy in Atlantic halibut (*Hippoglossus hippoglossus*) (23). Recently, a study exploring the genus *Takifugu*, revealed that three of the 12 species share a portion of the male-specific supergene containing *gsdf^Y^* as a candidate sex-determining gene (24). Furthermore, *gsdf* has been found to correlate with the expression of other sex-determining genes in medaka species (9, 25, 26) and in Atlantic salmon (*Salmo salar*) (27), indicating its involvement in male sex differentiation downstream from the initial sex determination.

Expression of this gonadal factor has been detected already at the larval stages in medaka species (9, 21, 26), zebrafish (*Danio rerio*) (28, 29), rainbow trout (16), Nile tilapia (*Oreochromis niloticus*) (30), and Japanese pufferfish (*Takifugu rubripes*) (31). In particular, *gsdf* mRNA was found in the gonadal primordium, within somatic cells surrounding the pre-meiotic germ cells. These findings indicate the involvement of *gsdf* in sex differentiation, likely associated with primordial germ cell (PGC) proliferation, as demonstrated in rainbow trout, where inhibition of *gsdf* translation resulted in a reduced number of PGCs (16). However, while sex reversal from male to female was observed in XY *gsdf*^-/-^ mutants of Japanese medaka (*Oryzias latipes*) and Nile tilapia (32–34), this phenotype was absent in zebrafish homozygous mutants, in which Gsdf appeared instead to regulate testis size without directly affecting spermatogenesis (28). These observations suggest that the role of Gsdf in male sex differentiation is not fully conserved among teleost lineages.

During juvenile development, Gsdf is involved in testis differentiation, showing a predominant gene expression in male over female gonads in several gonochoristic species, as documented in the review by Hsu and Chung (2021) (7). Similarly, in hermaphroditic species, *gsdf* expression is markedly upregulated during the transition to the testicular stage, suggesting an important role in the natural shift toward the male developmental pathway (35). The detection of *gsdf* mRNA in Sertoli cells surrounding the undifferentiated type A spermatogonia (SgA) points to a potential role in activation of germ cell proliferation, as demonstrated in juveniles of both gonochoristic and hermaphroditic teleosts (16, 36). After puberty, in adult specimens, the expression is restricted to the gonad, but the exact transcript and protein location, and the timing of expression may vary among species. Although exceptions have been reported, *gsdf* expression generally decreases as spermatogenesis progresses in males. Similar to the juvenile stage, its localization in Sertoli cells enclosing SgA links this gonadal factor to the promotion of germ cell self-renewal and proliferation (7).

The prominent role of this gonadal factor in testicular differentiation does not preclude its relevance in females related to ovarian development. Homozygous *gsdf* mutation in Japanese medaka (37), zebrafish (28), and Nile tilapia (15) causes ovarian hyperplasia and early-stage oocyte arrest with immature follicle accumulation, resulting in infertile females. These findings suggest that Gsdf is required for follicle maturation and oocyte growth, and therefore for female fertility.

The European sea bass is a marine fish of key economic and cultural importance in Europe (38) and an established model for basic research in reproductive endocrinology (39). It is a gonochoristic species lacking sex chromosomes with a temperature-influenced polygenic system that governs sex determination and differentiation (40, 41). Gonads remain undifferentiated during post-larval stages until 5–6 months of age, females differentiate earlier, reach bigger size, and mature later than males (42). Under intensive culture conditions, both sexes may enter puberty up to one year in advance, that is during the first or the second year of life for males or females, respectively. In our previous work investigating gonadal mechanisms driving sexual maturation of this species, the onset of precocious puberty was found to be negatively correlated with *gsdf1* expression in the testis, supporting its role as an early marker of puberty (18). Building on these observations and considering the remarkable diversity of teleost fish, the present study aims to examine the evolutionary relationship of the two European sea bass *gsdf* paralogues (*gsdf1* and *gsdf2*) in comparison with other species, explore their genomic context, and elucidate the transcriptional mechanisms regulating their expression. We provide a comprehensive characterization of the Gsdf paralogues in this species, at both gene and protein levels, with a focus on their roles during gonad differentiation, precocious male puberty and during the reproductive cycle of adult male and female gonads. For comparative purposes, *gsdf* homologues from other vertebrates were categorized as either *gsdf*, when displaying conserved synteny with European sea bass *gsdf1*, or *gsdf-like* when located in non-syntenic genomic regions. This framework enables a deeper understanding of the diversification and functional partitioning of *gsdf* genes in teleost reproduction.

## 2 Materials and methods

### 2.1 Comparative genomics and regulatory analyses

#### 2.1.1 Phylogenetic analysis and genomic synteny

The amino acid sequences used for alignments and phylogenetic analysis were extracted from Ensembl (Ensembl Genome Browser, http://www.ensembl.org; release v114, 05/2025) or NCBI (National Center for Biotechnology Information, http://blast.ncbi.nlm.nih.gov; 05/2025). The accession numbers are listed in **Supplementary Table 1**. Complete amino acid sequences were aligned using MUSCLE (Multiple Sequence Comparison by Log-Expectation) (43), and phylogenetic analysis was performed using the Molecular Evolutionary Genetics Analysis Software (MEGA12 (44)). The phylogenetic tree was constructed using the Maximum Likelihood method based on the Jones-Taylor-Thornton matrix-based model (45), based on the lowest Bayesian information criterion (BIC) score. A discrete Gamma distribution was used to model evolutionary rate differences among sites (6 categories (+ G, parameter = 2.245)). Gaps or missing data were pairwise deleted. One thousand bootstrapping replicates were used to assess the robustness of the inferred nodes of the tree. Percentage of amino acid sequence identity was calculated by using BLASTp (https://blast.ncbi.nlm.nih.gov/ (46)). Synteny analysis was performed by using the Genomicus tool (https://www.genomicus.bio.ens.psl.eu/genomicus-110.01/; release v110.1, 08/2021 (47)), based on Ensembl genome annotation. Representative species used for the analysis are listed in **Supplementary Table 1**; accession numbers of syntenic genes can be found in **Supplementary Table 2**.

#### 2.1.2 *In silico* analysis of upstream flanking sequences of *gsdf* genes

Analysis of the 5’ flanking regions of sea bass *gsdf1* and *gsdf2* genes was done as follows. Based on synteny analysis, 5 kb upstream sequences of the sea bass genes and those orthologues showing the highest conservation in the upstream region (presence of the two conserved neighboring genes) were extracted from Ensembl genome assemblies. Pairwise alignments were performed using the CHAOS/DIALIGN algorithm (48), to identify conserved non-coding sequences. The block with the highest conservation score, located proximal to the *gsdf1* transcription initiation, was selected for *de novo* motif discovery using MEME [Multiple Em for Motif Elicitation (49), implemented in the MEME Suite, http://meme-suite.org, version 5.5.8 (50)]. Analyses were run with parameters set for zero or one occurrence per sequence (ZOOPS) and a maximum of five motifs. Only motifs with E-value < 0.05 and alignment scoring > 5 were retained. These were subsequently analyzed with the Tomtom tool [Motif Comparison tool (51)] in the MEME Suite program for similarity to known transcription factor binding site in the JASPAR non-redundant DNA CORE 2024 vertebrate database [http://jaspar.elixir.no (52)]. Default parameters were used for motif comparison, including Pearson correlation coefficient as the similarity function, and a significance threshold for transcription factor identification was applied by setting the E-value <10.

### 2.2 Animal and tissue samples

Gonad samples from European sea bass at two key developmental windows were used for gene expression analysis: i) early juveniles (100–300 days post hatching, dph) including the gonad differentiation period; and ii) late juvenile males (350–430 dph), a period associated with the onset of precocious puberty in males. Both groups of samples were already available from previous works (53, 54). In brief:

- Early juveniles’ samples (53): specimens from 2 to 8 months after hatching underwent sequential size-grading, generating a small (70% males) and a large (97% females) population. Samplings were carried out every 50 days from 150 to 300 dph, and consisted of body trunks (150 dph), ventral wall of the swim bladder with the gonads attached (200 dph) and excised gonads (250 and 300 dph). Biometric parameters, sex differentiation and gonadal maturation stage in each sampling point have been already described (53). In the present work, aromatase gene expression (*cyp19a1a*) was used to ensure the sex of each sample (55).
- Late juvenile males (54): testis from 12-month old male sea bass were taken every 15 days from August (355 dph) to November (430 dph), covering the period for potential emergence of precocious puberty, recognized by the appearance of testis that undergo gametogenesis. At each sampling, only the largest 25% (potentially precocious males, N = 25) and the smallest 15% (immature males, N = 15) specimens were selected for the experiment. Additional specimens from both groups were kept until the end of the seasonal reproductive cycle (February) to establish the precocity occurrence (54).

Gonad samples were also obtained from 6-year-old adult European sea bass specimens (Males, total length = 51.20 ± 4.47 cm, total weight = 1996.81 ± 486.17 g; Females, total length = 54.98 ± 5.84 cm, total weight = 2498.74 ± 353.36 g) from a stock raised at the facility of Instituto de Acuicultura Torre de la Sal (IATS-CSIC, Spain, 40°NL) and maintained under natural photoperiod and temperature conditions. Sampling was performed monthly for an entire year (N = 5–6 fish/month/sex), covering a complete reproductive cycle in both sexes. Two sampling, early and late in the month, were carried out in September (onset of spermatogenesis) and November (onset of vitellogenesis), as these are key events in the reproductive cycle.

All experimental fish were anesthetized with an overdose of ethyl 3-aminobenzoate methanesulfonate salt (MS-222; 300–400 mg/L; Sigma-Aldrich^®^, St. Louis, MO, USA) and euthanized by decapitation, according to the Spanish (RD 53/2013) and European (Directive 2010/63/EU) regulations for the protection of animals used for scientific purposes. Specimens were dissected and gonads were partly frozen in liquid nitrogen and stored at -80 °C until use for RNA extraction, and partly stored at -20 °C until use for protein extraction. Small pieces of gonads were saved and processed for immunohistochemical detection (section 2.6), or used for histological analysis as follows: they were fixed by immersion in 4% formaldehyde: 1% glutaraldehyde (56), embedded in 2-hydroxyethyl methacrylate polymer resin (Technovit 7100, Heraeus Kultzer GmbH, Wehrheim, Germany), sectioned (2 µm) and stained as in Bennett et al. (1976) (57). The developmental stage of gonad was assessed following previously established criteria for males (58, 59) and females (59, 60).

RNA samples from 17 different tissues from adult males and females (N=2 pooled animals/sex) collected in November, were from a previous work (18) and were used for qPCR analysis.

cDNAs from isolated sea bass ovarian follicular cells, generated by Zapater et al. (2021) (61), were used for gene expression analysis, and frozen aliquots of those same cells were used for protein detection. In brief, these cells were isolated from ovaries of adult females in different maturation stages during a complete reproductive cycle, following a protocol previously described (62).

Gonadal tissues from 258 dph juvenile males and females (total weight = 48.36 ± 14.83 g) were used for protein detection by immunohistochemistry.

### 2.3 RNA isolation, cDNA synthesis and quantitative real-time RT-PCR

Gonadal tissue from adult gonads (15–20 mg) previously homogenized by Savant FastPrep FP120 (Cambridge Scientific, Watertown, MA, USA), were used for total RNA extraction, utilizing Maxwell™ 16 LEV simplyRNA Tissue Kit (Promega Corp., Madison, WI, USA) on a Maxwell™ 16 Instrument (Promega Corp.). The RNA was checked to be free of genomic DNA contamination and then used for cDNA synthesis: 1 µg of RNA was reverse transcribed using Superscript IV (Invitrogen Corp., Carlsbad, CA, USA) with random hexamers as primers, following the manufacturer’s instructions. As an internal control, 0.4 ng of mRNA from the *luciferase* (*luc1*) gene (luL4561, Promega Corp., Madison, WI, USA) were added to each reverse transcription reaction. When RNA was already available from previous works, its integrity and amount were checked and cDNA was directly synthesized as described above. Gene expression was analyzed by qPCR in 96-well plates with each sample in duplicate, and run in CFX384 Touch™ Real-Time PCR Detection System (Bio-Rad Laboratories, Inc., Hercules, CA, USA). Expression of the *luc1* was used as reference gene for data normalization, since it was stable among the different samples, except for gonads from 150–300 dph, where slight fluctuations were noted and a second housekeeping, the *18S* rRNA, was added. The geometric mean of the two housekeeping genes (*luc1*, *18S*) was used for the normalization in this set of samples.

The optimized amount of primers and probes (18, 61, 63) and the cDNA sample dilution used for each assay are shown in **Supplementary Table 4**. All qPCR components for *gsdf1* and *gsdf2* or for *luc1*, *18S*, and *cyp19a1a* were mixed with 5X PyroTaq EvaGreen Mix Plus No-ROX (CMB-Bioline, Madrid, Spain) or PyroTaq PROBE qPCR Mix Plus No-ROX (CMB-Bioline, Madrid, Spain), respectively, up to a final reaction volume of 20 μl.

For *luc1*, *18S*, and *cyp19a1a* fluorescence detection default settings were used, while for *gsdf1* and *gsdf2* the initial denaturation step for enzyme activation at 95 °C for 2 min was followed by 40 cycles of denaturation at 95 °C for 15 sec, annealing at 60 °C for 1 min and extension at 68 °C for 15 sec. Melting curves were determined by adding a dissociation step after the last amplification cycle, with 15 sec at 95 °C followed by an incremental increase from 60 °C to 95 °C in 0.5 °C every 15 sec. A single peak of melting curves was generated at the end of each reaction, confirming primers specificity. To correct for variability in amplification efficiency among different cDNAs and to quantify gene expression in *gsdf1*, *gsdf2*, *18S*, and *cyp19a1a* qPCR assays, appropriate standard curves were used, consisting of ten-fold serial dilutions of known concentrations of plasmids containing the target genes, obtained in previous works (18, 63). In the case of the reference gene *luc1*, the standard curve consisted of five-fold serial dilutions of a pool of cDNA samples. Data were captured and analyzed with CFX Manager™ Software (version 4.1). Correlation coefficients (R^2^) of the standard curves ranged between 0.972 and 0.998 and PCR efficiencies ranged from 70.1% to 118.7%.

### 2.4 Sea bass Gsdf-directed antibody

Rabbit antisera were produced on demand by Agrisera AB (Vännäs, Sweden). Anti-Gsdfs was raised against a synthetic peptide corresponding to amino acids C^206^EHGNIQQPSEE^217^ of sea bass Gsdfs, located in the C-terminal region. Due to the high similarity of Gsdf1 and Gsdf2 (87% of identity), the production of a specific antibody for each protein was not feasible, thus a single antibody was generated to detect both Gsdf1 and Gsdf2 proteins. The antibody was affinity-purified against the synthetic peptide used for immunization and its titer was tested by direct enzyme-linked immunosorbent assay.

### 2.5 Western blot analysis

Approximately 100 mg of each frozen gonad (stored at -20 °C) from adult sea bass males and females were transferred to 0.5 ml of cold radioimmunoprecipitation assay (RIPA) lysis buffer (25 mM Tris-HCl pH 7.5, 150 mM NaCl, 5 mM EDTA, 1% [v/v] Triton X-100, 0.5% [v/v] Sodium deoxycholate, 0.1% [v/v] SDS) containing protease inhibitors, i.e. 10 µg/ml leupeptin, 10 µg/ml aprotinin and 1 mM phenylmethylsulfonyl fluoride (PMSF) (all from Sigma-Aldrich^®^, St. Louis, MO, USA). Tissue was homogenized using FastPrep lysing matrix D (MP Biomedicals, CA, USA) on the Savant FastPrep FP120 instrument (Cambridge Scientific, Watertown, MA, USA), centrifuged at 12000 × g for 15 min at 4°C and finally the supernatant was recovered for protein analysis. Follicular cells were resuspended in cold RIPA lysis buffer in variable volumes depending on the initial cell number in each sample, such that 75 µl of buffer were used per 1 × 10^6^ cells. Cell lysis was performed by vortexing, followed by 10 min incubation on ice. Samples were centrifuged at 12000 × g for 15 min at 4°C and the supernatant was recovered. Total protein yields in testis, ovary and follicular cells were assessed in duplicates using Pierce BCA Assay (Thermo Fisher Scientific Inc., Waltham, MA, USA), according to the manufacturer’s protocol.

For immunoblotting, 70 µg of each protein extract were separated under denatured (95 °C, 5 min) and reducing conditions (5% 2-mercaptoethanol) by SDS-PAGE (12% resolving polyacrylamide tris/glycine gel) following standard procedures. After electrophoresis, proteins were transferred to previously activated 0.45 µm Immobilon-P-PVDF membranes (Millipore, Burlington, MA, USA) using the Trans-Blot Turbo™ Blotting System (Bio-Rad Laboratories, Inc., Hercules, CA, USA). The membranes were blocked overnight at 4°C in Tris-buffered saline with Tween 20 (10 mM Tris Base, 150 mM NaCl, 0.05% [v/v] Tween 20, pH 7.6) containing blotting-grade blocker (5% non-fat milk powder; Bio-Rad Laboratories, Inc., Hercules, CA, USA) and then incubated with the sea bass anti-Gsdf antibody (0.6 μg/mL) for 2 h at room temperature. Next, membranes were washed and further incubated with 1:25000-diluted goat anti rabbit immunoglobulin G horseradish peroxidase conjugate (GAR-HRP, Bio-Rad Laboratories, Inc., Hercules, CA, USA) for 1 h. Immunodetection was visualized by using enhanced chemiluminescence (Pierce™ ECL Plus Western Blotting Substrate, Thermo Fisher Scientific Inc., Waltham, MA, USA) in the Amersham Imager 600 (GE Healthcare Bio-Sciences, Chicago, IL, USA).

### 2.6 Immunohistochemical detection of endogenous Gsdf proteins

Gonadal tissues were fixed overnight at 4 °C in 4% paraformaldehyde (PFA) in phosphate-buffered saline (PBS), then dehydrated and embedded in paraffin. Sections of approximately 5 µm thickness were deparaffinized in xylene, rehydrated in decreasing concentrations of ethanol, and washed twice with double-distilled water. Slides underwent heat-induced antigen retrieval in Tris-EDTA Buffer (10 mM Tris Base, 1 mM EDTA, and 0.05% Tween 20, pH 9.0) at 95 °C for 15 min and subsequently were cooled down at room temperature. Two washes were performed with Tris-buffered saline 0.1% Triton X-100 (TBS-T), then samples were blocked with TBS-T 3% Normal Goat Serum (NGS), 1% Bovine Serum Albumin (BSA) for 2 h, and finally incubated overnight at 4 °C with 1.2 µg/mL of anti-Gsdf primary antibody in TBS-T 3% NGS, 1% BSA (primary antibody stock [600 µg/mL]; dilution factor 1:500)). The day after, sections were washed twice in TBS-T, immersed in TBS 0.5% hydrogen peroxide for 20 min to quench endogenous peroxidase activity, and then incubated for 1.5 h at room temperature with secondary antibody (GAR-HRP, Bio-Rad Laboratories, Inc., Hercules, CA, USA) diluted 1:200 in TBS-T 3% NGS, 1% BSA. After three washes in TBS-T, slides were treated during 3–5 min with 3,3′-Diaminobenzidine (DAB, ACROS Organics, Waltham, MA, USA) used as substrate for color development. Nuclei were counterstained with 25% hematoxylin (Sigma-Aldrich ^®^,St. Louis, MO, USA) for 10 s. Slides incubated without primary antibody served as a negative control. Sections were examined and photographed by a Nikon Eclipse E600 imager microscope (Nikon Instruments, Europe BV, Kingston, Surrey, England).

### 2.7 Statistical analysis

Data are shown as the mean ± standard error of mean (SEM) and all statistical analysis and graphical representations for qPCR data were performed by using GraphPad Prism version 8.1 (GraphPad Software, Inc., La Jolla, CA, USA). Before proceeding with comparative analyses among experimental groups, the normality of each dataset was tested. The outliers, if present, were excluded from analysis. Differences in the temporal profile of *gsdf1* and *gsdf2* in European sea bass juveniles (150–300 dph) were analyzed by two-way ANOVA, one-way ANOVA and Student’s t-test. In particular, two-way ANOVA was performed to analyze the effect of age and sex/size/precocity occurrence on the gene expression and the interaction between these factors. One-way ANOVA, followed by *post-hoc* Tukey’s test, was performed into each group to delve into the temporal profile expression in males and females; Student’s t-test was used to compare groups at each developmental age, to assess sex differences. The *gsdf1* and *gsdf2* expression profile in testis, ovary and follicular cells from adult specimens during the annual reproductive cycle was analyzed by Kruskal-Wallis non-parametric test (ANOVA on ranks), followed by *post-hoc* Dunn’s multiple comparison test (testis and ovary) or one-way ANOVA, followed by *post-hoc* Tukey’s test (follicular cells). In all tests, differences were accepted as statistically significant starting from *p* < 0.05. The Kruskal-Wallis non-parametric test, followed by *post-hoc* Dunn’s multiple comparison test, were used to account for temporal differences in gene expression because normality requirements were not fulfilled.

## 3 Results

### 3.1 Comparative genomic and regulatory analysis

#### 3.1.1 Phylogenetic and syntenic analysis

**Figure 1:**
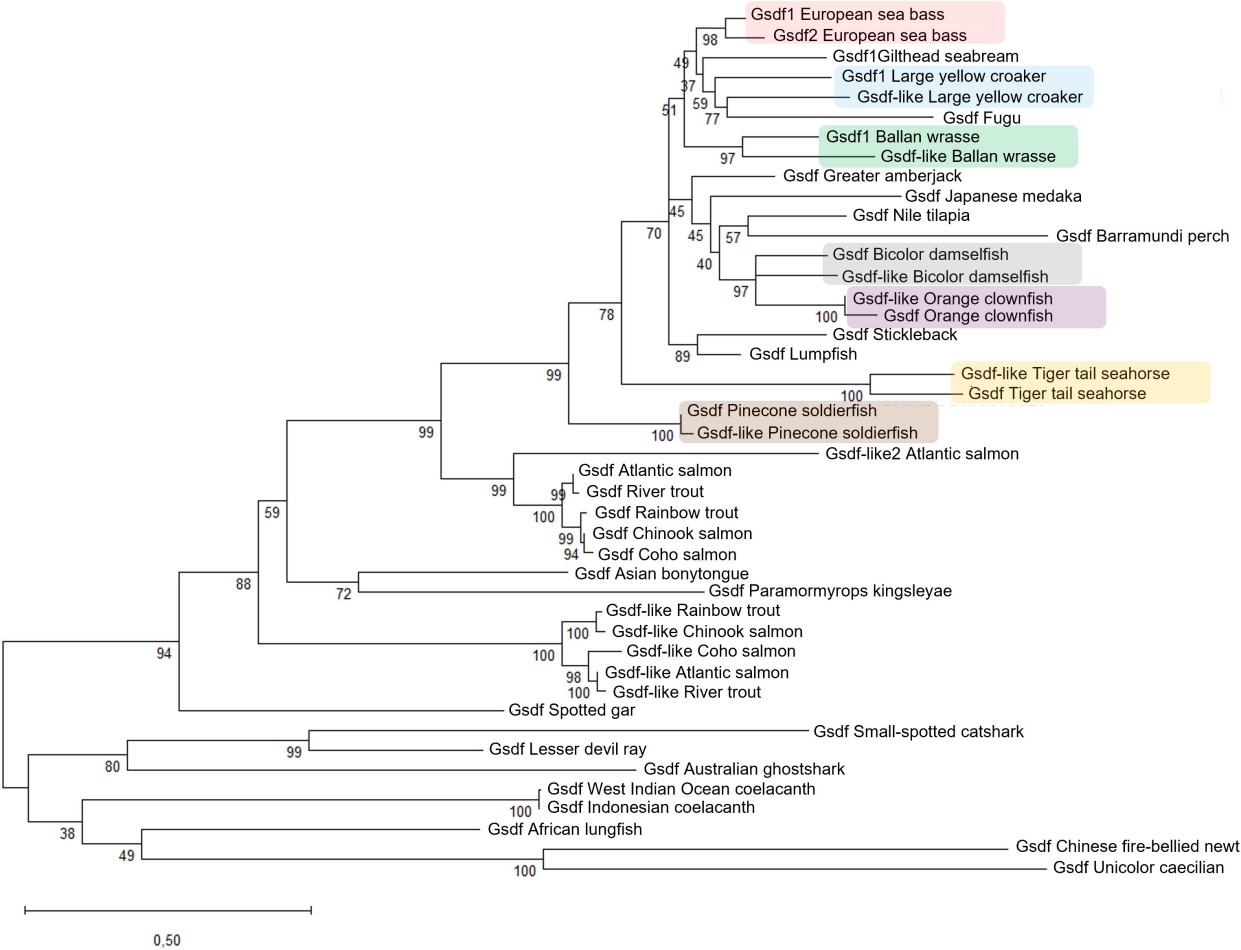
Rooted phylogenetic tree of Gsdf protein sequences from 31 vertebrate species. The tree was built using the Maximum Likelihood method based on the Jones-Taylor-Thornton matrix-based model and SPR extensive search. Bootstrap support values (1,000 replicates) are shown at the nodes as percentage. Intraspecific gene duplications are visually highlighted by colored rectangles. Ensembl or NCBI accession numbers of the sequences are indicated in the **Supplementary Table 1**.

A phylogenetic tree of *gsdf* genes was generated by the Maximum Likelihood method to illustrate the evolutionary relationship among 31 different vertebrate species, 12 of which harbor *gsdf* duplicates, resulting in 44 sequences (**Supplementary Table 1**). The tree revealed two main clades (**Figure 1**), supported by high bootstrap values, indicating strong robustness of the inferred relationships. The first clade comprised *gsdf* genes from chondrichthyans, basal sarcopterygians and early-diverging amphibians. The second and larger clade was rooted in spotted gar (*Lepisosteus oculatus*) and divided in two subclades, one grouping *gsdf-like* genes from salmonids, and the other encompassing all *gsdf* orthologues and duplicates from all the other species used in the present study. Within this latter subclade, Asian bonytongue (*Scleropages formosus*) and *Paramormyrops kingsleyae*, which are basal teleosts, branched separately, while the largest branch consisted of two sub-groups, e.g., *gsdf* genes from Salmonids, and *gsdf* genes from all the other teleosts. A closer inspection on the phylogenetic tree within this latter well-supported sub-group revealed that species mainly belong to Percomorpha, a large and diverse clade of ray-finned fish considered the most recently evolved teleost group. In this branch, species appeared distributed according to their evolutionary relationship, with European sea bass clustering alongside gilthead seabream (*Sparus aurata*), large yellow croaker (*Larimichthys crocea*), fugu (*Takifugu rubripes*) and ballan wrasse (*Labrus bergylta*).

As highlighted by the colored boxes (**Figure 1**), seven Percomorph species possess two copies of *gsdf* that cluster as species-specific sister pairs. In contrast, salmonid *gsdf* duplicates cluster into two distinct groups, with one copy being more divergent from other fish *gsdf* than the other. These phylogenetic findings are consistent with the amino acid identity found in intra- (between paralogs) and inter-specific (between orthologs) comparisons of Gsdf proteins (**Supplementary Table 3**). In most Percomorphs, sequence identity between paralogs within a species was higher than that obtained when each paralog was compared with European sea bass orthologue. Two exceptions were observed: in rainbow trout (a salmonid), where intra-specific identity was markedly low (40.70%), and in large yellow croaker, where the two paralogs were more similar to their respective European sea bass orthologues (Gsdf 70.70%; Gsdf-like 67.28%) than to each other (62.91%).

**Figure 2:**
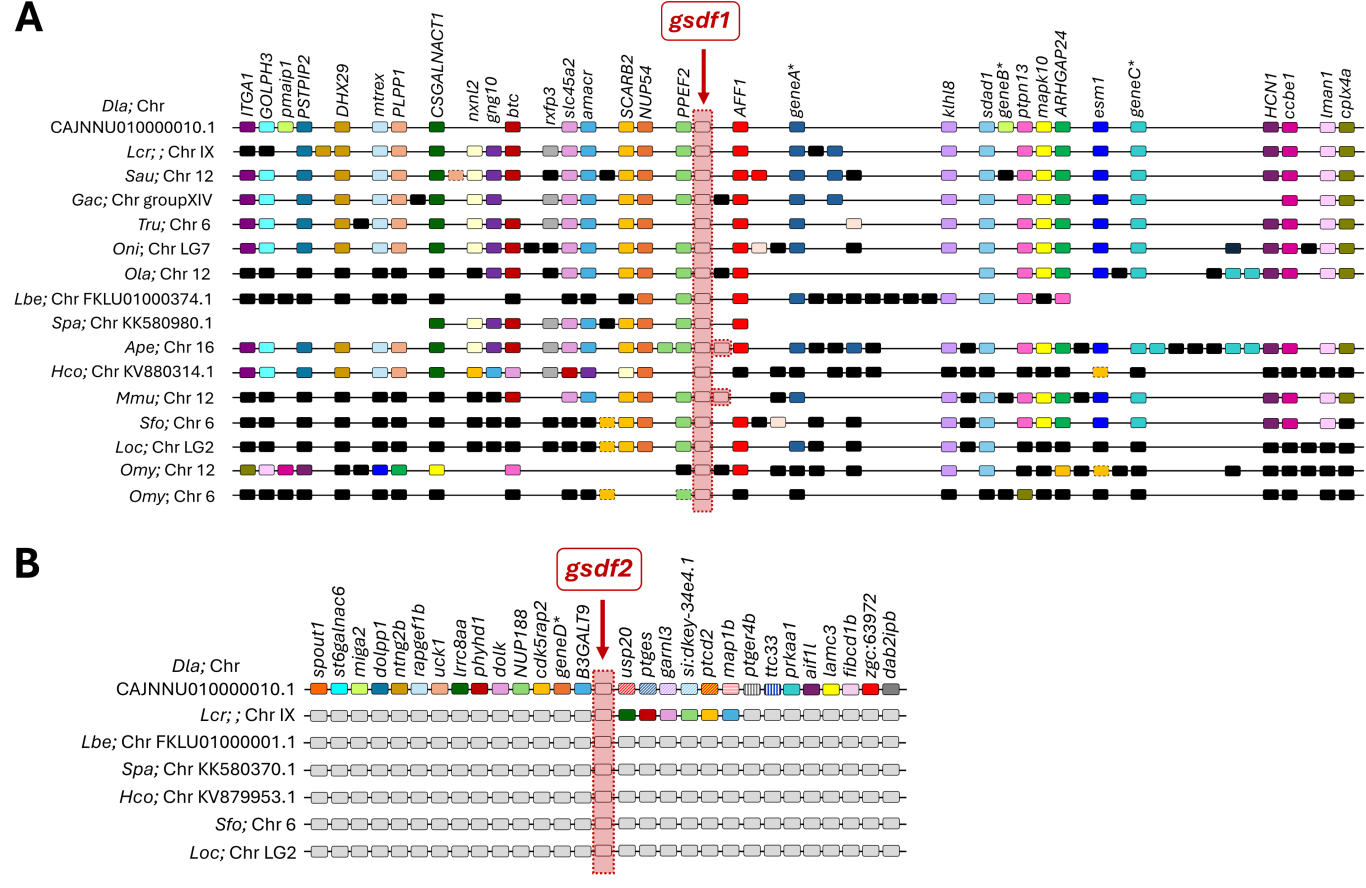
Syntenic alignment of the chromosomic regions around **(A)***gsdf* (*gsdf1* in European sea bass) and **(B)***gsdf-like* (*gsdf2* in European sea bass) genes in different teleost species. The synteny was analyzed with Genomicus v110.01 using the European sea bass genes as reference. Chromosome segments are represented by black lines, colored rectangles denote individual genes, with orthologs indicated by identical colors. Paralogues are represented by same color but dashed border. The red vertical square highlights *gsdf* orthologues in different species. Black **(A)** or grey **(B)** squares represent non-orthologous genes, indicating a lack of synteny. Genes in the flanking regions were retrieved from Ensembl and the abbreviations are listed in **Supplementary Table 2**. *gsdf* orthologues (listed in **Supplementary Table 1**) were extracted from Ensembl genome assemblies of European sea bass (*Dla*) (*Dicentrarchus labrax*), large yellow croaker (*Lcr*) (*Larimichthys crocea*), gilthead seabream (*Sau*) (*Sparus aurata*), stickleback (*Gac*) (*Gasterosteus aculeatus*), fugu (*Tru*) (*Takifugu rubripes*), Nile tilapia (*Oni*) (*Oreochromis niloticus*), Japanese medaka (*Ola*) (*Oryzias latipes*), ballan wrasse (*Lbe*) (*Labrus bergylta*), bicolor damselfish (*Spa*) (*Stegastes partitus*), orange clownfish (*Ape*) (*Amphiprion percula*), tiger tail seahorse (*Hco*) (*Hippocampus comes*), pinecone soldierfish (*Mmu*) (*Myripristis murdjan*), Asian bonytongue (*Sfo*) (*Scleropages formosus*), spotted gar (*Loc*) (*Lepisosteus oculatus*), rainbow trout (*Omy*) (*Onchorynchus mykiss*).

Synteny analysis were performed to investigate the conservation of the genomic context of sea bass *gsdf1* and *gsdf2*, used as reference genes, and 14 genes both upstream and downstream were considered (**Supplementary Table 2**). Representative species from each clade were selected from the phylogenetic tree (**Supplementary Table 1**), including all teleosts with duplicates (*gsdf* and *gsdf-like)*. To explore the potential conservation of ancestral genomic arrangements and distinguish them from teleosts-specific features, spotted gar and Asian bonytongue were also incorporated in the synteny analysis. The genomic region flanking *gsdf1* in European sea bass showed high conserved synteny with orthologous regions in five teleost species, large yellow croaker, gilthead sea bream, stickleback (*Gasterosteus aculeatus*), fugu and Nile tilapia, indicating the presence of a highly conserved block (**Figure 2A**). In contrast, the ballan wrasse *gsdf* region displayed a markedly lower synteny, with only two and five genes in the upstream and downstream regions of *gsdf*, respectively. In the same line, Japanese medaka genome harbored only six genes from the conserved upstream block, and two genes located near *gsdf1* in the downstream region are also missing.

Among species with duplicated *gsdf* genes, both orange clownfish (*Amphiprion percula*) and pinecone soldierfish (*Myripristis murdjan*) exhibited the two duplicates adjacent to each other in the chromosome, on the same and on the opposite strands, respectively. Synteny in these species was highly conserved, particularly in the orange clownfish, while in tiger tail seahorse (*Hippocampus comes*) and bicolor damselfish (*Stegastes partitus*) only the *gsdf1* upstream region showed high similarity with that of sea bass (**Figure 2A**). Synteny became increasingly lost in more distantly related species, in Asian bonytongue, conservation was notably diminished in the upstream region, whereas in spotted gar synteny was disrupted on both flanking regions, retaining only 3–4 conserved genes per side (**Figure 2A**).

In rainbow trout, two *gsdf* genes are present, known as *gsdf1* in Chr. 6 (16) and *gsdf2* in Chr 12 (17). Rainbow trout *gsdf2* was surrounded upstream and downstream by some genes located downstream of sea bass *gsdf1*, while rainbow trout *gsdf1* retained only two orthologs upstream and one downstream, indicating a higher syntenic divergence (**Figure 2A**). Based on the synteny analysis and their situation in the phylogenetic tree, rainbow trout *gsdf1* and *gsdf2* were named as *gsdf-like* and *gsdf*, respectively, in the present study.

European sea bass *gsdf1* and *gsdf2* loci did not share any neighboring gene on both upstream and downstream sides, indicating no conserved synteny (**Figures 2A, B**). So, potential syntenic conservation was explored among sea bass *gsdf2* genomic context and the *gsdf-like* genes from other teleosts, which had not shown syntenic conservation with sea bass *gsdf1*. Genomic regions from Asian bonytongue and spotted gar *gsdf* were also included. Partially conserved synteny was only found with large yellow croaker: six genes located upstream of *gsdf2* in sea bass were retained downstream of *gsdf-like* locus in croaker (**Figure 2B**). In both sea bass and croaker genomes, their *gsdf* duplicates were located in the same chromosome, in different strands, separated by 57 and 356 genes, respectively (**Supplementary Figure 1A**). A more detailed analysis of the *gsdf2* genomic regions in sea bass and croaker revealed the presence of differently rearranged small conserved blocks (**Supplementary Figure 1B**).

#### 3.1.2 Analysis of regulatory motifs in the 5’ flanking sequences of *gsdf* genes

The 5’ flanking regions of *gsdf* genes were analyzed to assess the existence of conserved regulatory sites among teleosts and 10 species were selected for this purpose (**Supplementary Table 1**).

First, the pairwise alignment of 5 kb regions located upstream of European sea bass *gsdf1* and *gsdf2* revealed the presence of a large conserved block close to the transcription start site (TSS), corresponding to -168 respect to the TSS for *gsdf1* (-259 respect to the ATG start codon) and -224 respect to the TSS for *gsdf2* (-245 respect to the ATG start codon). In this block, four motifs with high alignment score were identified (**Supplementary Figure 2A**). In light of these findings, the promoter region of sea bass *gsdf2* was aligned with both the sea bass *gsdf1* promoter and the orthologous regions from the other 9 selected teleosts.

**Figure 3:**
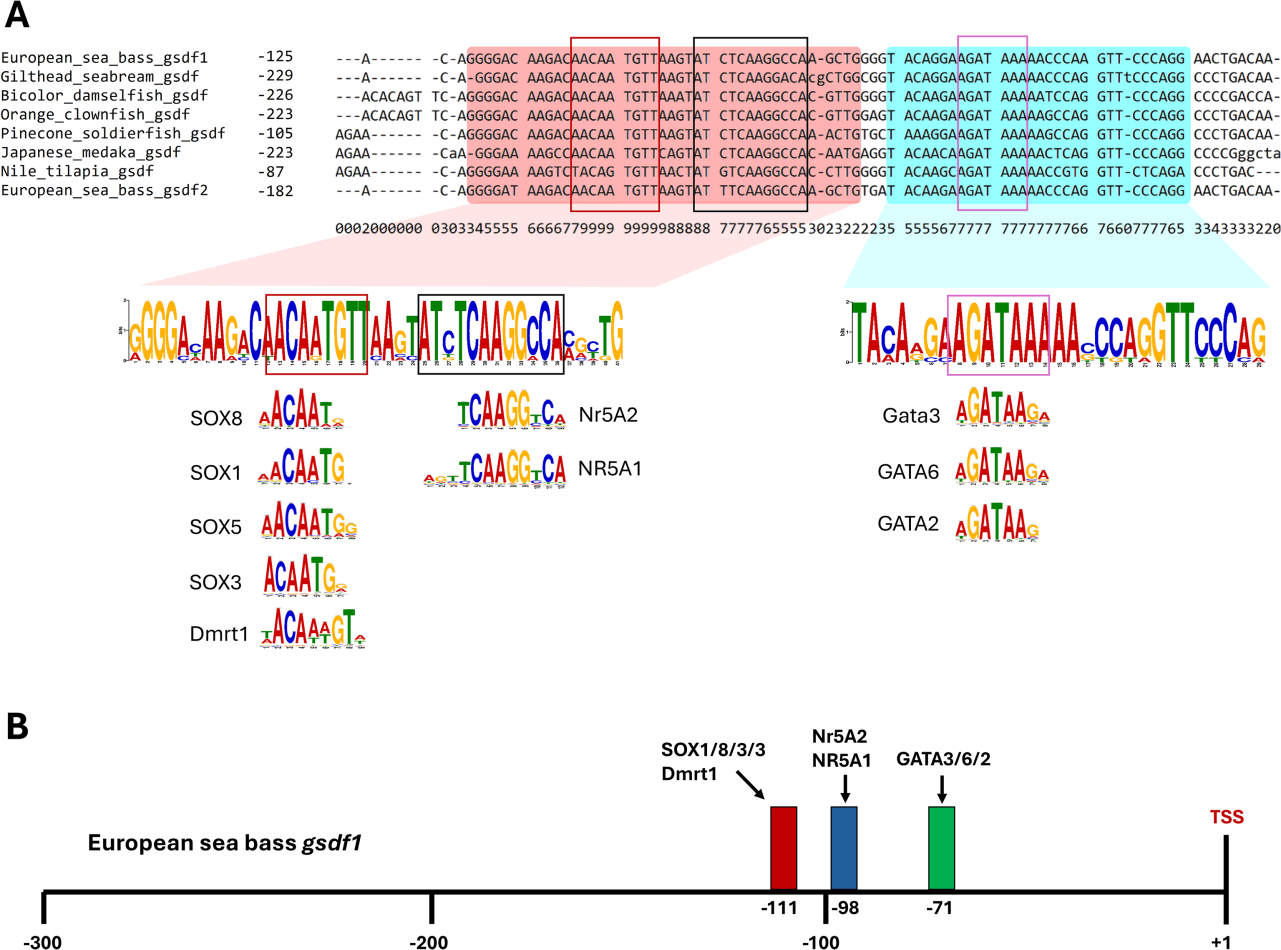
Analysis of the 5’ flanking region of *gsdf*. **(A)** Proximal promoter regions of the *gsdf* genes identified in European sea bass (*Dicentrarchus labrax*), gilthead seabream (*Sparus aurata*), bicolor damselfish (*Stegastes partitus*), orange clownfish (*Amphiprion percula*), pinecone soldierfish (*Myripristis murdjan*), Japanese medaka (*Oryzias latipes*), Nile tilapia (*Oreochromis niloticus*) genomes were aligned using the CHAOS DIALIGN algorithm. Conserved motifs were identified by the MEME tool in MEME Suite program and highlighted by colored rectangles. Evolutionary conserved DNA motifs are marked with boxes. Binding sites for known transcription factors were predicted using the Tomtom tool (MEME Suite) based on comparisons with the JASPAR 2024 non-redundant CORE vertebrate database. SOX: SRY-related HMG-box containing proteins; Dmrt1: Doublesex and Mab-3 Related Transcription Factor 1; Nr5a1 and Nr5a2: Nuclear Receptor Subfamily 5 Group A Members 1 and 2; GATA: transcription factors of the GATA family. **(B)** Schematic representation of the putative cis-regulatory elements located on the *gsdf1* proximal promoter.

Consistently, the pairwise alignment of 5 kb of genomic sequences revealed the presence of a large conserved block in 7 out of the 10 species analyzed (**Figure 3A**). This block showed a high conservation score and was located immediately upstream to the transcription start site (TSS), within a region ranging from -322 to -101 bp, respect to the TSS of each species (**Figures 3A, B**). Five motifs with statistically significant E-value, indicating evolutionary sequence conservation across species, were identified within this conserved block (**Supplementary Figure 2B**). Among them, two motifs exhibited alignment scores >5 and were conserved among the seven species (**Figure 3A**). The comparison of these motifs against the JASPAR vertebrate database identified three sites in which different transcription factor may bind (**Figure 3B**). Specifically, using *gsdf1* promoter region as reference, putative binding sites for SOX1, SOX3, SOX5, SOX8, and Dmrt1were found at -111 bp respect to the sea bass *gsdf1* TSS (**Figures 3A, B**). Within the same motif, at -98 bp, binding sites for Nr5A1 and NR5A2, also known as SF-1 and LRH-1, respectively, were identified (**Figures 3A, B**). Finally, the binding sites for GATA2, Gata3, and GATA6 were found at -71 respect to the sea bass *gsdf1* TSS (**Figures 3A, B**).

### 3.2 Tissue distribution of *gsdf1* and *gsdf2* mRNA

**Figure 4:**
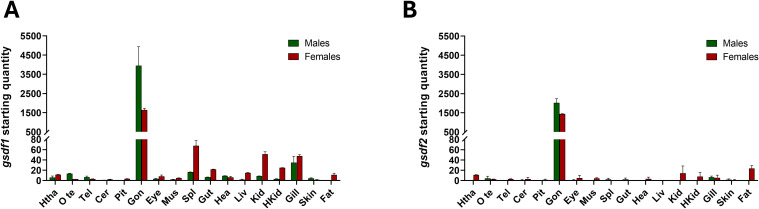
Tissue distribution of *gsdf1***(A)** and *gsdf2***(B)** expression detected by qPCR. Adult specimens (2 pooled animals/sex) were sacrificed in November. Green bars represent male sea bass tissues; red bars female sea bass tissues. Tissues: Htha, hypothalamus; O te, optic tectum; Tel, telencephalon; Cer, cerebellum; Pit, pituitary gland; Gon, gonad; Eye; Mus, muscle; Spl, spleen; Gut; Hea, heart; Liv, liver; Kid, kidney; HKid, head kidney; Gill; Skin; Fat. Data are reported as mean ± SEM.

The expression of *gsdf1* and *gsdf2* was evaluated by qPCR in different tissues from adult sea bass of both sexes at the beginning of their reproductive cycle (November) (**Figure 4**). Gene expression showed a similar pattern in males and females, with markedly higher levels in gonads of both sexes. In males, *gsdf1* was more highly expressed than *gsdf2* in all samples; it showed maximal expression in testis, moderate expression in gills, very low levels in all other tissues and was undetectable in fat (**Figure 4A**). In contrast, *gsdf2* was nearly undetectable in all tissues except in testis, where the maximum expression was detected, but at lower levels than *gsdf1* (**Figure 4B**). In females, both *gsdf1* and *gsdf2* were most abundantly expressed in the ovary compared to other tissues (**Figures 4A, B**). Expression of *gsdf1* was found in all female tissues, being more evident in spleen, kidney and gill, and lower in others (**Figure 4A**); *gsdf2* showed low expression in fat and kidney, and almost undetectable in all other tissues (**Figure 4B**).

### 3.3 Expression of *gsdf1* and *gsdf2* during gonad differentiation

Given the specific gonadal expression of *gsdf1* and *gsdf2* in both sexes, a more detailed analysis focused on the gonads was conducted across the lifespan of European of sea bass.

In early juveniles aged 150–300 dph, gene expression was assessed in male- (small) or female- (large) dominant populations. The phenotypic sex for each sample was further ensured by the expression levels of *cyp19a1a* gene, high for females and low for males (55) (**Supplementary Figure 3**), except for 150 dph gonads, in which *cyp19a1a* values were similar for all specimens, relying then in the *small* (presumptive males) or *large* (presumptive females) classification. In juveniles between 150 and 300 dph, age primarily reflects distinct stages of gonadal development rather than a chronological effect. Each sampling point is therefore critical to capture the progression of gonadal differentiation and its impact on gene expression.

**Figure 5:**
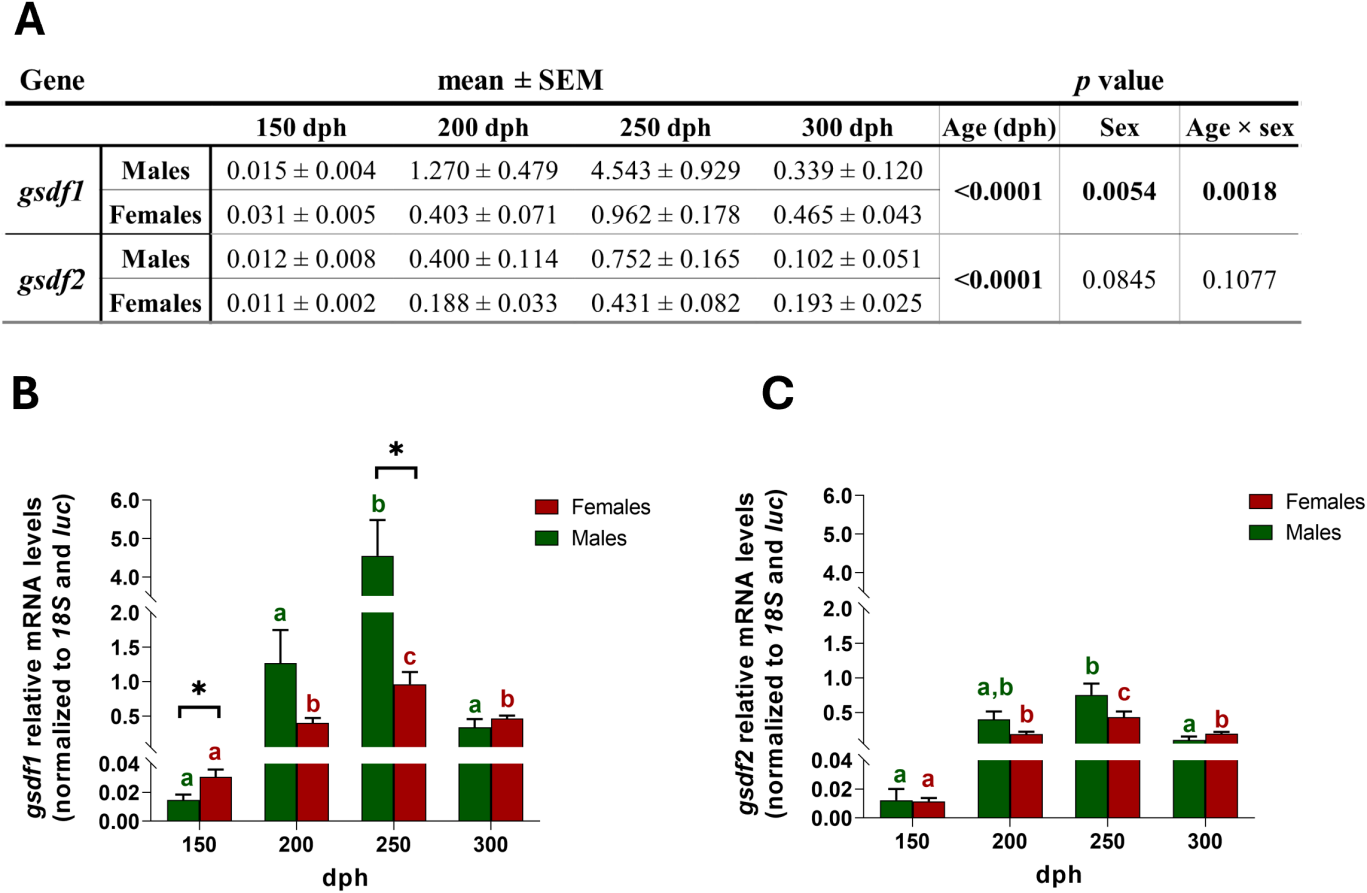
Temporal profile of *gsdf* mRNA levels in early juveniles of European sea bass. Two-way ANOVA analysis **(A)**, *gsdf1***(B)** and *gsdf2***(C)** expression in juveniles of both sexes during gonad development from 150 to 300 dph (N = 63). Data are reported as mean ± SEM. Two-way ANOVA was performed to analyze the effect of age and sex on gene expression and the interaction between these factors. One-way ANOVA, followed by *post-hoc* Tukey’s test, was performed into each group, males or females; Student’s t-test was used to compare sexes at each developmental age. Letters represent statistical significance (*p* < 0.05) among time points within an experimental group, asterisk indicated statistical difference (*p < 0.05) between sexes at each time point.

Differential *gsdf1* expression was observed in relation to sex and age (gonadal developmental stage) and their interaction (**Figure 5A**), revealing distinct temporal expression profiles in males and females during and after gonadal differentiation. In males, levels were very low at 150 dph, gradually increased by 200 dph, reached the maximum pick at 250 dph (significantly higher than all other time points), and sharply declined by 300 dph (**Figure 5B**). Females also showed low expression at 150 dph, followed by a significant rise at 200 dph, a further increase at 250 dph and a drop at 300 dph (**Figure 5B**). Although both sexes exhibited a similar trend, the magnitude differed markedly: at 250 dph, males showed a 300-fold increase relative to 150 dph, compared with an approximately 32-fold increase in females, resulting in levels 4-fold higher in males than in females. Significant sex differences were also detected at 150 dph, with higher values in females, although expression was minimal in both sexes (**Figure 5B**). The expression of *gsdf2* displayed exactly the same trend that *gsdf1*, with a gradual increase with age in both sexes peaking at 250 dph, but with markedly lower levels than *gsdf1*, about 4-fold lower in males and 2-fold lower in females (**Figure 5C**). Statistical analysis revealed significant variation of *gsdf2* expression with age (gonad developmental stage), whereas neither sex nor the interaction between factors was significant (**Figure 5A**). In fact, males and females exhibited similar *gsdf2* levels at all age points, with no significant sex differences (**Figure 5C**).

### 3.4 Expression of *gsdf1* and *gsdf2* in puberty onset in males

Following the developmental gene expression analysis, the focus shifted to males, as expression was generally higher than in females. In 1-year-old European sea bass, only the largest males acquire functional competence of the reproductive axis and enter puberty precociously. This situation provides an opportunity to compare gene expression between fish of the same age but at different reproductive maturation status. To this end, *gsdf1* and *gsdf2* expression was examined in gonads from two size-based groups of males (“small” or “large”, 355–440 dph, N = 69) from a previous experiment (54). By the end of the trial (February), no specimens from the small group had reached spermiation and were classified as immature, whereas 75% of the large group were spermiating males (Stage V) (54), demonstrating that larger fish tend to reach puberty at the end of the first year and could be considered precocious.

**Figure 6:**
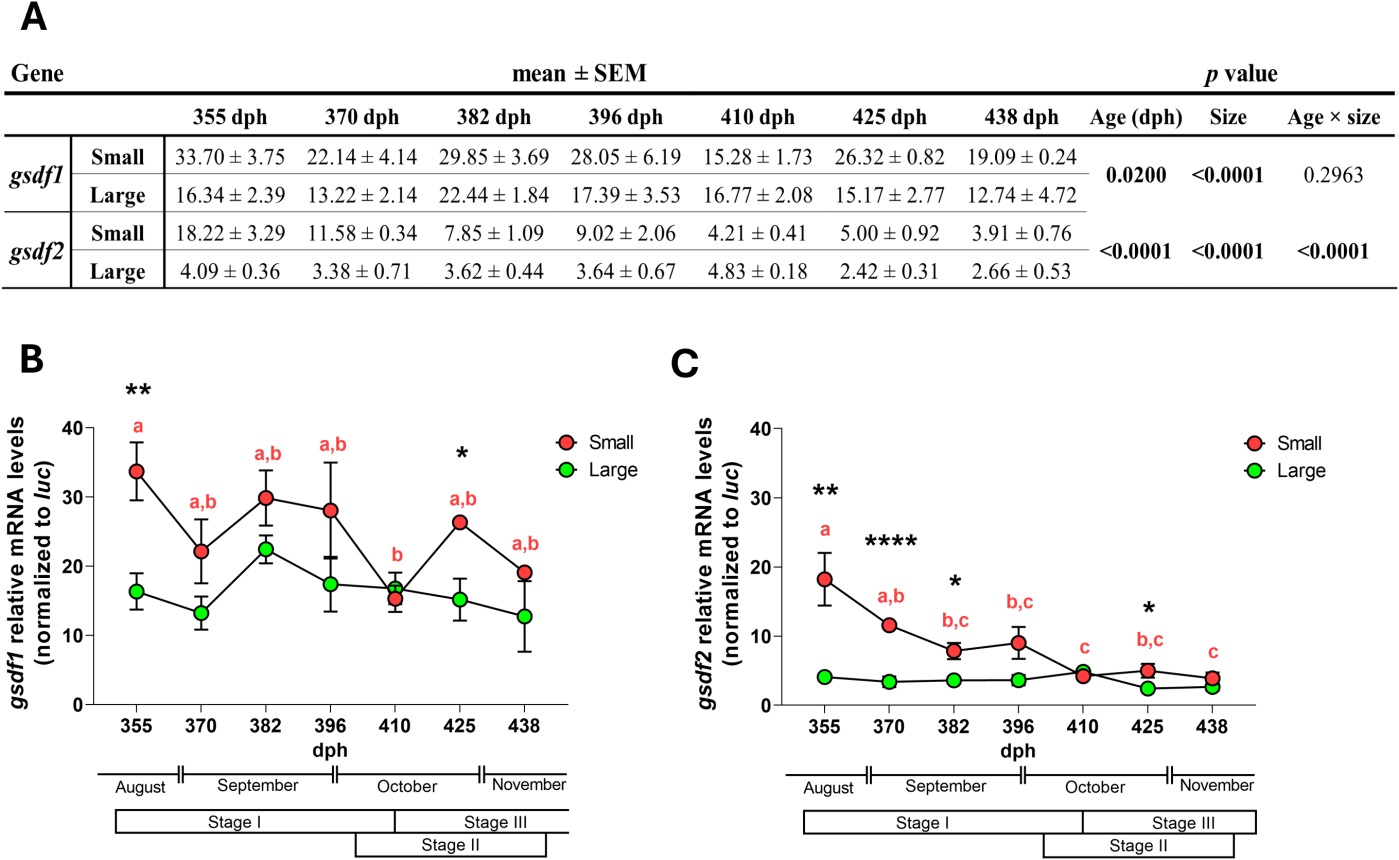
Temporal profile of *gsdf* mRNA levels in male juveniles of European sea bass. Two-way ANOVA analysis **(A)**, *gsdf1***(B)** and *gsdf2***(C)** in male juveniles (N = 69) divided into two groups according to the size/precocity occurrence (small=immature, N = 34; large=precocious, N = 35). Data are reported as mean ± standard error of mean (SEM). Two-way ANOVA was performed to analyze the effect of age and size/precocity occurrence on gene expression and the interaction between these factors. One-way ANOVA, followed by *post-hoc* Tukey’s test, was performed into each group; Student’s t-test was used to compare groups at each developmental age. Letters represent statistical significance (*p* < 0.05) among time points within an experimental group, asterisks indicate statistical difference (*p < 0.05; **p < 0.01; ****p < 0.001) between groups at each time point.

Both age and size/precocity significantly influenced *gsdf1* and *gsdf2* expression (**Figure 6A**). In this trial, “age” (355–440 dph) did not primarily reflect a chronological effect but rather a critical period (August-November) of the reproductive cycle, coinciding with the onset of spermatogenesis in precocious males (large group). For *gsdf1*, no significant interaction between these two factors was observed, whereas *gsdf2* expression varied with age in a size-dependent manner.

The expression pattern of *gsdf1* was similar in both groups, with comparable fluctuations throughout the experimental period (**Figure 6B**). The main factor influencing *gsdf1* expression was size/precocity status: immature (small) males consistently showed higher expression levels than precocious (large) males, with significant differences in August, before the onset of spermatogenesis in the precocious group, and again in late October, after spermatogenesis had begun in these animals (**Figure 6B**).

In large precocious specimens, *gsdf2* expression remained consistently low, with no significant changes across the trial. In contrast, immature specimens presented significantly higher *gsdf2* expression, peaking in August and then gradually declining to a minimum in October-November (**Figure 6C**). At most sampling points, *gsdf2* expression differed significantly between the two groups. Overall, *gsdf2* was expressed at lower levels than *gsdf1* in both groups, and precocious males (large group) exhibited lower expression of both genes compared with immature specimens.

### 3.5 Seasonal changes of *gsdf1* and *gsdf2* expression in adult gonads

Considering the dimorphic expression of *gsdf1* and *gsdf2* observed in juvenile males and females, as well as in immature or precocious testes, the expression analysis was extended to adult gonads throughout an annual reproductive cycle. **Supplementary Figures 4A**, **5A** illustrate in detail the monthly percentage distribution of maturity stages for testis and ovary, respectively. Gonadal phases were assigned according to histological criteria (58–60) and representative photomicrographs are included in **Supplementary Figures 4C-H**, **5C-H**. The reproductive season takes place during the winter months (Nov-Mar), when both sexes reach their highest gonadosomatic index (GSI) values (**Supplementary Figures 4B**, **5B**).

**Figure 7:**
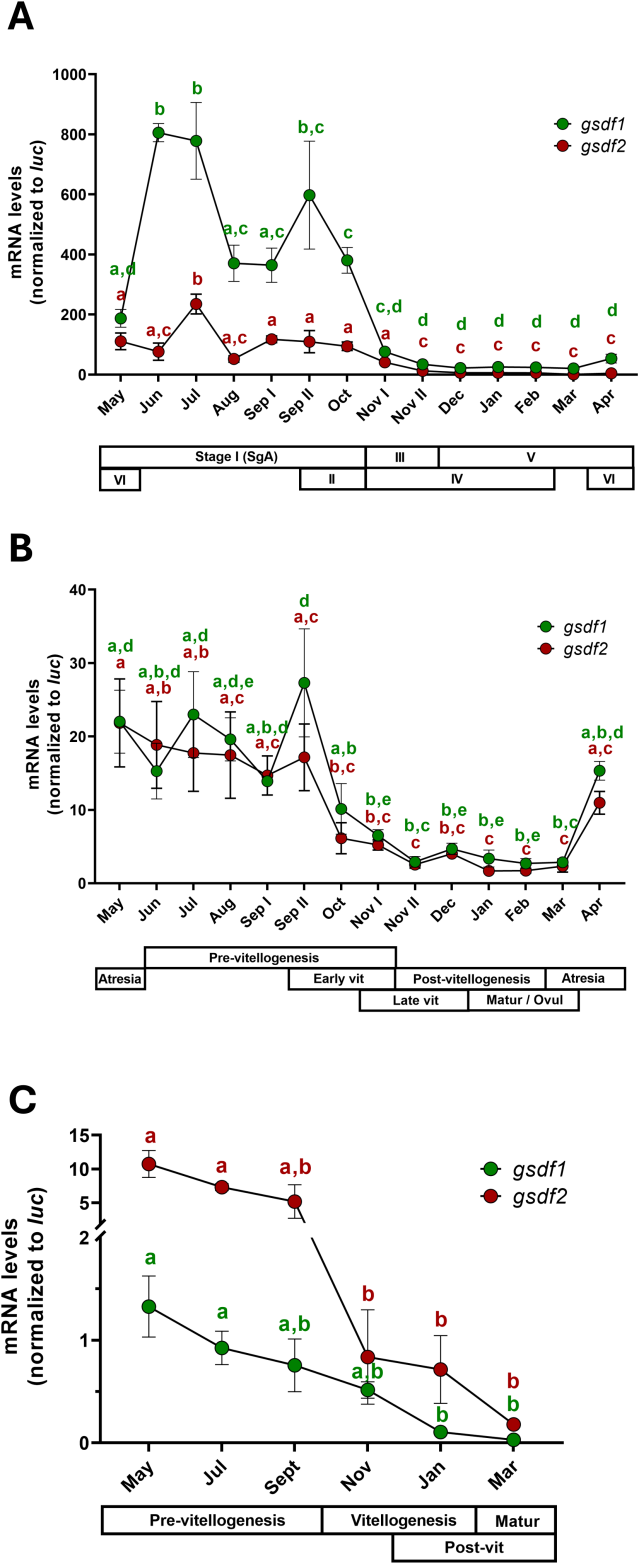
Temporal expression of *gsdf1* and *gsdf2* in testis **(A)**, ovary **(B)** and ovarian follicular cells **(C)** from 6-year-old adult European sea bass specimens during an annual reproductive cycle. N = 5–6 fish/month/sex were used for gene expression analysis in gonads, N = 17 females were used to isolate follicular cells. Data are reported as mean ± standard error of mean (SEM) and analyzed by Kruskal-Wallis Kruskal-Wallis test, followed by post-hoc Dunn’s test (**(A)** testis and **(B)** ovary) or one-way ANOVA, followed by *post-hoc* Tukey’s test (**(C)** follicular cells). Letters represent statistical significance (*p* < 0.05) among time points within each experimental group.

In males, *gsdf1* consistently predominated over *gsdf2*, reaching expression levels up to 4-fold higher (**Figure 7A**). Both genes showed significantly increased transcriptional activity from May to October, corresponding to the pre-meiotic stage of testis (Stage I), with peaks in June-July. Expression dropped sharply at the beginning of November, coinciding with the onset meiosis and progression of gametogenesis, and remained at minimal levels until April (**Figure 7A**).

In females, *gsdf1* and *gsdf2* were expressed at similar levels, displaying parallel temporal patterns. Transcript abundance was highest during the pre-vitellogenic stage, from May to September, and declined significantly from October to March, when vitellogenesis progressed toward maturation/ovulation (**Figure 7B**). Notably, a sharp increase in *gsdf1* and *gsdf2* expression was detected in April, at the end of the spawning season, preceding the rise observed in males in June (*gsdf1*) or July (*gsdf2*).

When male and female expression profiles were compared, both genes exhibited strong male-biased expression, with *gsdf1* up to 20-fold and *gsdf2* up to 10-fold higher in testes than in ovaries (**Figures 7A, B**). Interestingly, analysis of isolated ovarian follicular cells revealed a markedly different pattern compared to the whole ovary tissue: *gsdf2* expression was nearly 10-fold higher than *gsdf1* (**Figure 7C**). Consistent with ovarian expression, the highest transcript levels of both genes in follicular cells were detected between May and September, corresponding to atretic and pre-vitellogenic ovaries.

### 3.6 Detection of endogenous Gsdf proteins in adult sea bass gonads

Gsdf affinity purified anti-sera was validated against concentrated culture media of CHO cells transiently transfected with expression plasmids for *gsdf1* or *gsdf2* (pcDNA3-*gsdf1* or pcDNA3-*gsdf2*) (**Supplementary Material** and methods) and protein extracts from sea bass testes and ovaries. A distinct band at approximately 25 kDa was detected in both gonad extracts and concentrated CHO culture medium (**Supplementary Figure 6**), consistent with the predicted molecular weight of the protein (23.384 kDa).

**Figure 8:**
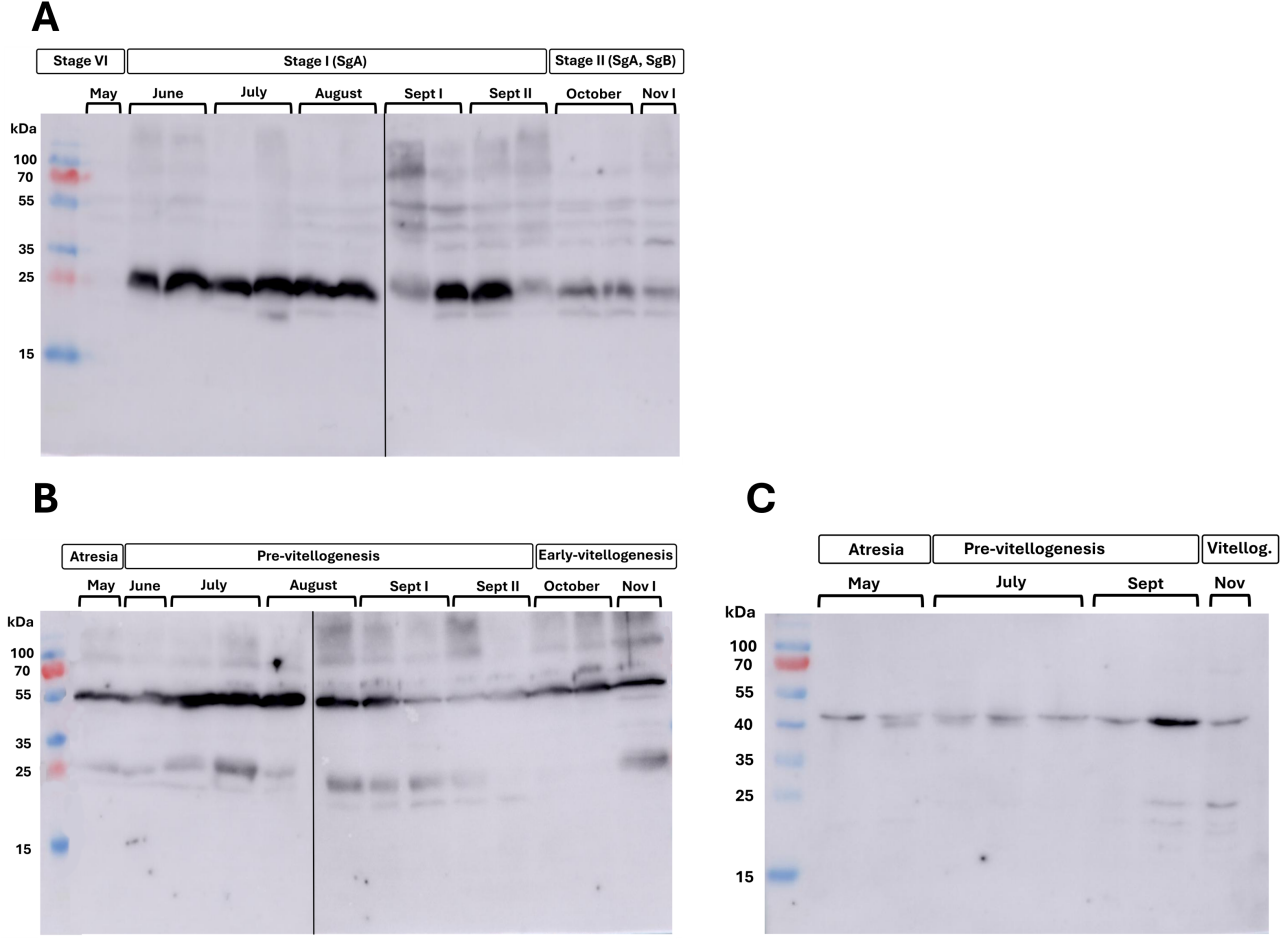
Western blot detection of Gsdf proteins in gonad extracts (70 µg total protein/lane) from adult European sea bass. Testes **(A)**, ovaries **(B)**, and isolated ovarian follicular cells **(C)** during the early-mid gametogenesis (May-November). One or two extracts per month were chosen as representative.

The presence of sea bass Gsdfs was assessed in extracts from adult gonads collected during the months of highest gene expression (May-November) by Western Blot analysis. In testis extracts, a predominant band of ~25 kDa was detected. Band intensity revealed that these proteins were nearly absent in May, strongly present during the summer months (from June to August/September), and progressive declined from late September onward, as indicated by weaker bands observed in the autumn months (October–November) (**Figure 8A**).

In the ovarian extracts, two bands were recognized, one at ~25 kDa, similar to males, and another, consistently more intense, at ~55 kDa. Proteins were detected in all sampled months; but band intensities were stronger in July and August, suggesting a higher abundance of these proteins in the gonads during this period (**Figure 8B**). Similarly, in extracts from isolated follicular cells the antibody detected a main band at ~50 kDa throughout the analyzed period and a fainter one at ~25 kDa during the onset of vitellogenesis (September-November) (**Figure 8C**).

### 3.7 Localization of endogenous Gsdf proteins in pre-puberal and adult male and female sea bass

**Figure 9:**
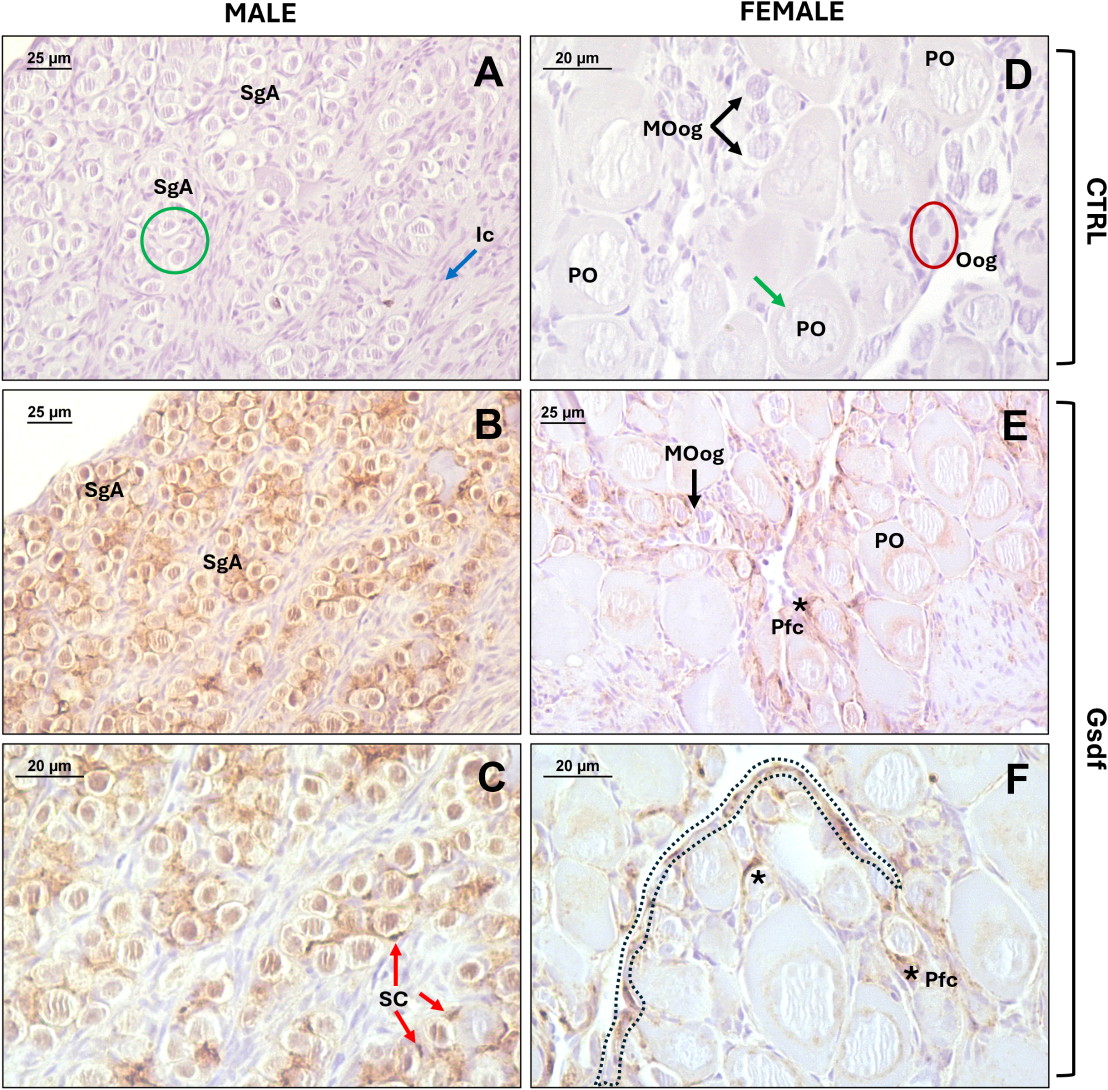
Immunohistochemical localization of sea bass Gsdf proteins in immature gonads from juvenile specimens (258 dph). **(A-C)** Photomicrographs from males, showing immature testes characterized by SgA surrounded by Sertoli cells and organized in lobules defined by interstitial cells. **(D-F)** Photomicrographs from females, showing immature ovaries characterized by an active germinal epithelium containing oogonia and primary oocytes; primary follicles are also seen in the lamellae. **(A, D)** Control sections without primary antibody; **(B, C, E, F)** Gsdf staining signal in section incubated with 1.2 µg/mL of anti-Gsdf primary antibody. SgA, type A spermatogonia (green circle); SC, Sertoli cells surrounding SgA (red arrows); Ic, Interstitial cells (blue arrows); Oog, oogonia (red circle); Moog, oogonia entering meiosis I (black arrows); PO, primary oocytes (green arrows); Pfc, pre-follicular cells (asterisks); germinal epithelium (dashed black line). Bars = 20 µm **(C, D, F)**, 25 µm **(A, B, E)**.

Cellular localization of Gsdf1 and Gsdf2 was assessed in gonads of prepubertal juveniles of European sea bass, given the high expression of the *gsdf* genes at that stage (**Figure 5**). In juvenile males, whose testes were entirely composed of type A spermatogonia (SgA) organized in cysts, the signal (brown color) was clearly detected in Sertoli cells (SC) surrounding SgA (**Figures 9B, C**), in comparison with the control sections (**Figure 9A**), and in nucleus and perinuclear area of SgA. Females displayed a differentiated ovary, in which oogonia (Oog), oogonia entering meiosis I (Moog), and primary oocytes (PO) could be distinguished in the germinal epithelium. Oogonia are in contact with undifferentiated somatic cells that form the germinal epithelium, and when entering meiosis begin to be surrounded by a thin layer of pre-follicular cells (Pfc). Gsdf proteins were detected along the germinal epithelium, and also in pre-follicular cells surrounding newly formed oocytes that are still inside the germinal epithelium. In more developed primary oocytes, already located within the ovarian lamellae and with a proper follicular structure, Gsdf was detected inside the oocyte, specifically in the perinuclear region, likely corresponding to the endoplasmic reticulum (**Figures 9E, F**).

**Figure 10:**
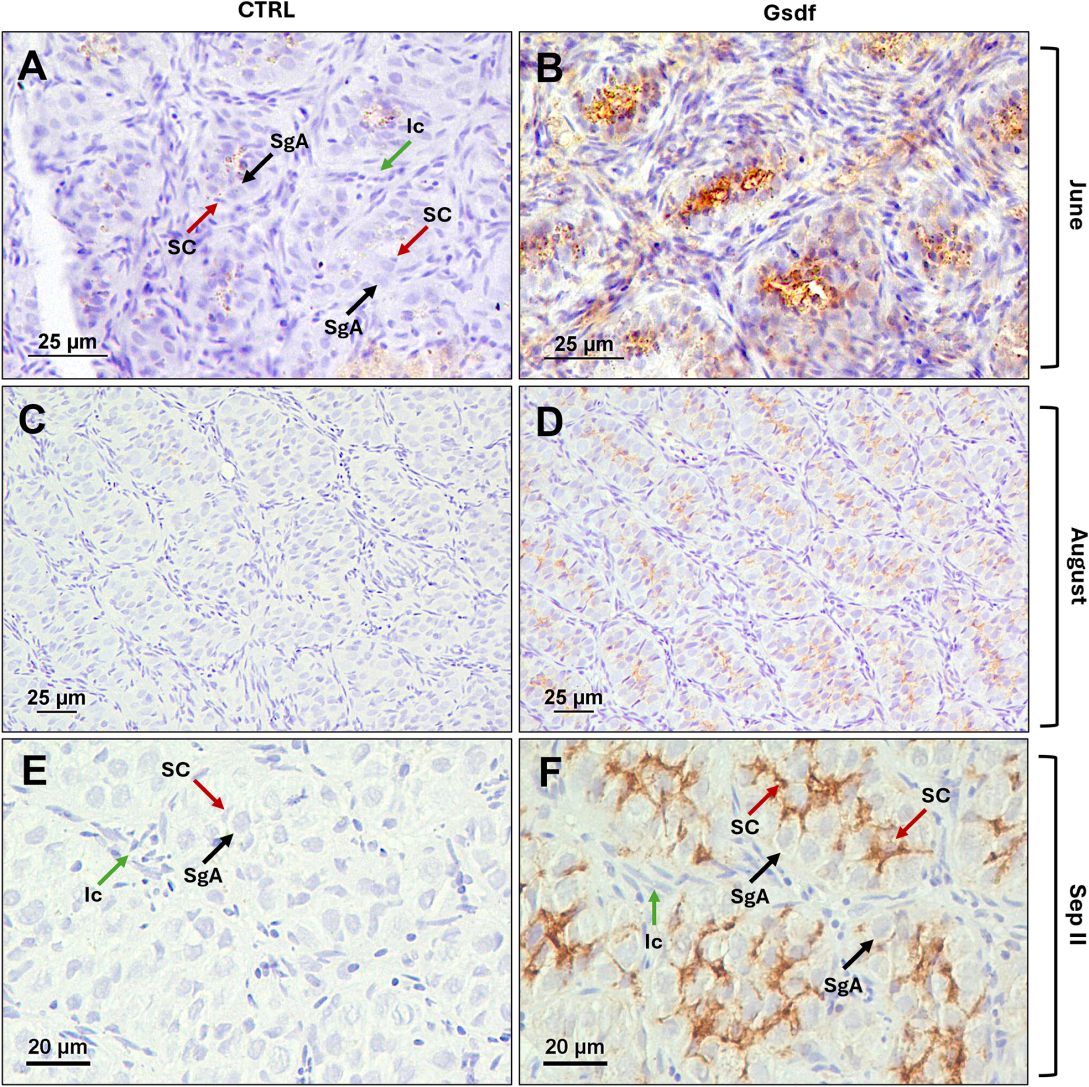
Immunohistochemical localization of sea bass Gsdf proteins in pre-meiotic testis from adult specimens. Photomicrographs from June **(A, B)**, August **(C, D)**, and September II **(E, F)**, showing immature stage characterized by type A spermatogonia surrounded by Sertoli cells and organized in lobules defined by interstitial cells. **(A, C, E)** Control sections without primary antibody; **(B, D, F)** Gsdf staining signal in section incubated with 1.2 µg/mL of anti-Gsdf primary antibody. SgA, type A spermatogonia (black arrows); SC, Sertoli cells surrounding SgA (red arrows); Ic, Interstitial cells (green arrows). Bars = 25 µm **(A-D)**, 20 µm **(E, F)**.

In adult gonads, representative months exhibiting strong Gsdf detection by Western blot were selected for cellular localization by immunohistochemistry. In males, testis samples from June to September were selected. These correspond to testes in pre-meiotic stage, characterized by SgA organized in lobules. In June, just two months after the end of the spawning season, the testes show a high proportion of interstitial tissue and lobules still contain few germ cells (SgA), which will proliferate during the summer and in early Autumn, most of them will commit to differentiation and enter spermatogenesis. The presence of the Gsdf proteins was confirmed, as all sections incubated with anti-Gsdf primary antibody showed staining signal (brown color) (**Figures 10B, D, F**) in comparison to the control section (**Figures 10A, C, E**). Gsdf was immunodetected in Sertoli cells enclosing SgA; interestingly, the signal was exclusively observed in the central area of the lobule, with no detection around peripheral spermatogonia (**Figures 10B, D, F**).

**Figure 11:**
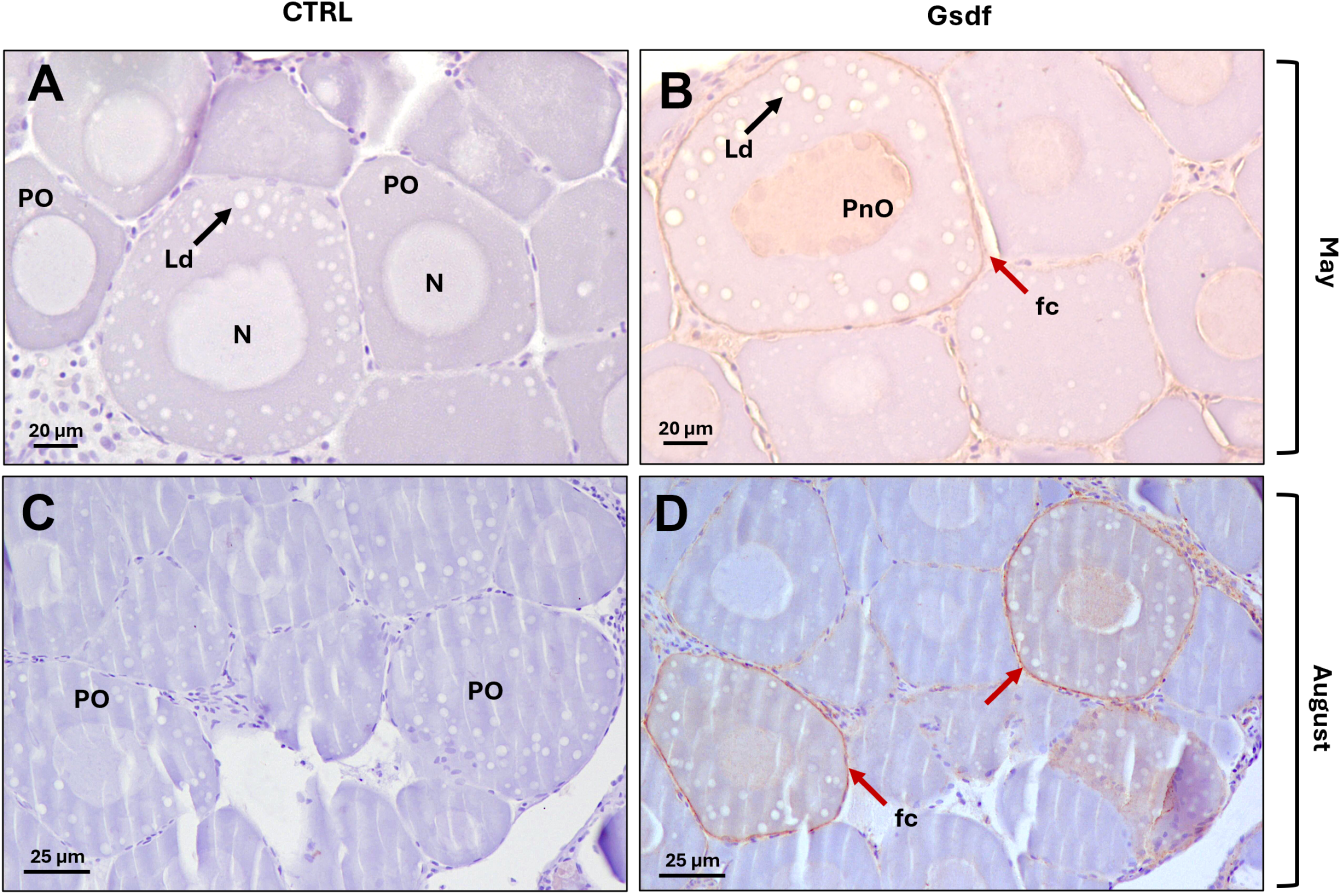
Immunohistochemical localization of sea bass Gsdf proteins in pre-vitellogenic ovary from adult specimens. Photomicrographs from May **(A, B)**, and August **(C, D)**, showing pre-vitellogenic follicles characterized by primary oocytes, some exhibiting lipid droplet accumulation and multiple nucleoli distributed along the inner nuclear membrane. **(A, C)** Control sections without primary antibody; **(B, D)** Gsdf staining signal in section incubated with 1.2 µg/mL of anti-Gsdf primary antibody. PO, primary oocytes; N, nucleus; Ld, lipid droplets (black arrows); PnO, perinuclear oocytes; fc, follicular cells (red arrows). Bars = 20 µm **(A, B)**, 25 µm **(C, D)**.

In females, representative ovaries from May and August, corresponding to the pre-vitellogenic stage, were selected. Gonads were entirely composed of primary oocytes, some exhibited more advanced features, such as multiple nucleoli located at the periphery of the nucleus (perinuclear oocytes) and the initial accumulation of lipid droplets in the cytoplasm. Compared to the control sections (**Figures 11A, C**), Gsdf staining (brown color) was clearly evident in follicular cells surrounding primary oocytes containing oil droplets, and slight staining was also observed inside those oocytes, whereas no signal was detected in less advanced oocytes (**Figures 11B, D**). In August a faint signal was also detected in the already very thin germinal epithelium.

## 4 Discussion

The present study provides a comprehensive characterization of the two *gsdf* paralogs identified in the European sea bass, integrating evolutionary, genomic, and expression analyses at both gene and protein levels. Phylogenetic reconstruction and synteny comparison revealed that the duplication of *gsdf* in this species is not linked to the teleost-specific whole genome duplication (3R), but instead represents an independent event that has occurred in other teleost lineages as well. The two paralogues exhibit different sex-specific expression suggesting sub-functionalization in sea bass: *gsdf1* is predominantly expressed in males, particularly during early testicular development, while *gsdf2* shows higher expression in females, especially in follicular cells during the pre-vitellogenic phase.

For a long time, the *gsdf* was considered a teleost-restricted gene. However, whole genome sequencing in the last decade revealed its presence in basal sarcopterygians, showing that *gsdf* is not exclusive to teleosts. First identified in coelacanth (*Latimeria menadoensis*) (64), bona fide *gsdf* sequences were subsequently found in West African lungfish (*Protopterus annectens*) and in Australian ghostshark (*Callorhinchus milii*) (11), suggesting its presence in the common ancestor of *Chondrichthyes* and *Osteichthyes*. More recently, *gsdf* was identified for the first time in a tetrapod, in the amphibian Chinese fire-bellied newt (*Cynops orientalis*, now named *Hypselotriton orientalis*) (8), indicating the retention of this gene in the sarcopterygian ancestors of tetrapods. In all these species, *gsdf* displayed a male-biased expression pattern, suggesting an evolutionarily conserved role in testicular development. The origin of *gsdf* has been linked to the second whole genome duplication event, given its absence in primitive lineages such as sea lamprey (*Petromyzon marinus*) (65) and it has been suggested that *gsdf*, *bmp15* and *gdf9* arose as paralogs with a subsequent loss of a fourth ohnologue (28).

In the present study, the phylogenetic relationships of *gsdf* were explored across 31 different species. In accordance with the above-mentioned works (11, 64), this gene is retained in *Chondrichthyes* and in basal *Sarcopterygii*, including *Actinistia* (coelacanths) and *Dipnoi* (lungfish). Moreover, in our phylogenetic tree, the sarcopterygian branch also included two early-diverging amphibians, but not Xenopus, a more recently diverged species which lacks *gsdf*. This pattern indicates that *gsdf* was retained in an ancient tetrapod ancestor, and lost at least twice in the evolutionary history of tetrapods, in the Anuran lineage and in the common ancestor of Amniota, explaining its absence in reptiles, birds, and mammals (8).

Bonytongues formed the earliest teleost branch after the teleost-specific whole genome duplication (66), with the remaining species grouped in a larger clade. Seven out of 16 species exhibited *gsdf* duplications that clustered together as sister sequences, denoting independent, lineage-specific duplication events unrelated to the 3R duplication. To further investigate the origin and evolutionary dynamics of these intra-specific duplicates, a synteny analysis was conducted, revealing a more complex evolutionary scenario than initially expected. In European sea bass and several other species, *gsdf* duplicates lacked conserved intra-specific synteny, pointing to duplication mechanisms other than simple tandem duplication, such as transposon-mediated or ectopic events, which are common in teleost genomes (67–69). European sea bass and large yellow croaker showed similar dynamics, with a duplication event followed by genomic rearrangement, resulting in the two paralogues being located in the same chromosome (**Supplementary Figure 1A**). In the orange clownfish and pinecone soldierfish, the duplicated genes are adjacent with high sequence identity, likely originated from recent tandem duplication. In contrast, in ballan wrasse, bicolor damselfish, and tiger tail seahorse the *gsdf* paralogs are on different chromosomes, supporting a segmental duplication. All these findings reinforce the hypothesis that *gsdf* is located in a polymorphic genomic region, subject to a high frequency of chromosomal inversions, rearrangements, and gene duplications.

Despite the instability of this genomic context, the flanking regions of European sea bass *gsdf1* showed a remarkable degree of conserved synteny across teleost species, including more basal representatives such as Asian bonytongue, and the non-teleost ray-finned fish spotted gar. This region contains evolutionarily conserved genes in teleosts, including *nup54*, *aff1*, *klhl8*, *ptpn13*, and *sdad1*, which are mainly expressed in ovaries of medaka and zebrafish, particularly in pre-vitellogenic oocytes (12, 29). Interestingly, these loci are also present in the genomes of chicken (*Gallus gallus*) and human (*Homo sapiens*) (12, 29, 65, 70), as shown in our updated *in silico* analysis (**Supplementary Figure 7A**), indicating that *gsdf* was lost in most tetrapods, while the surrounding genes within the same syntenic block were retained (7).

Differently from *gsdf* genes, no teleost *gsdf-like* duplicate retained any conserved synteny with sea bass *gsdf1* or *gsdf2*, further confirming that they are orthologues originated independently with species-specific dynamics. The only exception was the *gsdf-like* of large yellow croaker, which exhibited partial synteny with sea bass *gsdf2*, reflecting comparable duplication histories. The similarity between these two species in the *gsdf* duplicates’ dynamics is consistent with previous studies, which highlighted the evolutionary proximity between sea bass and croaker (71, 72). Notably, in the synteny analysis of sea bass *gsdf2*, the use of the neighboring *nup188* gene as reference markedly increased conservation (**Supplementary Figure 8**). This result indicates that, although the *gsdf2* locus itself may have undergone lineage-specific rearrangements, it is embedded within a conserved syntenic block retained across teleosts and tetrapods, as several of the surrounding genes are also present in human and chicken genomes (**Supplementary Figure 7B**).

In contrast to the aforementioned *gsdf* duplicates, salmonid *gsdf* paralogues formed two monophyletic clades in the phylogenetic tree, consistent with the salmonid-specific 4th whole genome duplication (Ss4R), and prior to *Salmo* - *Oncorhynchus* divergence (73). Analysis in rainbow trout, where *gsdf1* and *gsdf2* are well characterized (16, 17), revealed that *gsdf2*, but not *gsdf1*, shared the highest synteny with *gsdf1* from sea bass and other teleosts, leading to their designation as *gsdf-like* (*gsdf1*) and *gsdf* (*gsdf2*). The same pattern observed in rainbow trout was consistently found across other salmonid species, suggesting lineage-specific genomic rearrangements, as shown by their identical syntenic context of both *gsdf* and *gsdf-like* (**Supplementary Figure 9**).

The evolutionary and genomic conservation of *gsdf* genes observed in this study suggests functional or regulatory constraints. Comparative analysis of the putative promoter regions of selected teleost *gsdf* genes, including sea bass *gsdf1* and *gsdf2*, revealed conserved transcription factor binding sites across species. A Dmrt1 binding site was predicted, consistent with previous findings in Nile tilapia (34), sablefish (22), gibel carp (19), and spotted scat (*Scatophagus argus*) (74). The involvement of Dmrt1 in reproductive processes is well documented (75). It is a key regulator of male development in teleosts (76–79), and a duplicated copy of *dmrt1* acts as a master sex-determining gene in Japanese medaka (80). It has been described in several fish species that Dmrt1 and Steroidogenic Factor 1 (Sf1, also known as Nr5a1) interact to activate *gsdf* transcription in a dose-dependent manner (19, 34, 74). Both genes are expressed in testis (10, 74, 81), specifically in testicular somatic cells (10, 19, 34), and are critical for male differentiation, as demonstrated in medaka, where double *gsdf/dmrt1* mutants underwent irreversible male-to-female sex reversal (32).

The sea bass *gsdf* promoters also contain binding sites for Nr5a1 and/or Nr5a2, paralleling observations in zebrafish (29), spotted scat (74), and gibel carp (19). In mammals, *NR5A1* and *NR5A2* promote gonadal somatic cell reprogramming and regulate gonadal development in both sexes (82–85). In teleosts, duplication events have resulted in distinct paralogous copies of these genes, resulting in *ff1b* and *ff1d* co-orthologues of *NR5A1* and *ff1a* orthologue of *NR5A2* (18, 86). A potential interaction between *gsdf* and *ff1b* was already suggested in our previous work, in which both genes appeared negatively correlated with the onset of puberty in European sea bass males (18). *Nr5a1* promotes ovary development upregulating *cyp19a1a* in several teleosts (87–90), while *Nr5a2* mimics *Nr5a1* in follicular differentiation (88, 91) or performs “pro-male” functions (92, 93), depending on the species.

Putative binding sites for SOX family members (Sox1, Sox3, Sox5, and Sox8) were also identified. SOX family factors play crucial roles in several fish physiological processes, including reproduction (94). While Sox1 is primarily involved in neurogenesis, both in mammals (95, 96) and in teleosts (97, 98), Sox5 contributes to the formation of multiple tissues and organs in fish (94), acting in sex determination as a regulator of germ cell number (99) and in male sex differentiation (100). Sox3 and Sox8 play roles primarily in sex determination/differentiation and gonadal development. Sox3, expressed by somatic and germ cells, is mainly associated to oogenesis and ovary differentiation (101, 102), although in some species it plays important roles also in males (94, 103), acting as a master sex-determining gene in Indian medaka (*Oryzias dancena*) and up-regulating *gsdf* expression (25). Sox8 is crucial for adult Sertoli cell function and male fertility in mammals (104, 105), and is consistently involved in testis development in fish (106, 107), besides, additional roles have been reported in some fish species (94).

Additionally, GATA factors (Gata2, Gata3 and Gata6) binding sites are conserved in teleost *gsdf* promoters, as also found in zebrafish (29). Gata2 regulates multiple functions in teleosts (108, 109), and shows ubiquitous tissue expression, with a predominance in testis over ovary in tongue sole (*Cynoglossus semilaevis*) (110). In contrast, Gata3 is associated with the immune system and gill development (111, 112). Finally, Gata6 plays an important role in embryonic morphogenesis and in gonadal functions, with male-biased expression in somatic and germ cells (113, 114). In light of the above, the present study demonstrated that teleosts retain several regulatory elements in the *gsdf* promoter that are, primarily but not exclusively, involved in reproductive functions.

Focusing on European sea bass, our comprehensive analyses of *gsdf1* and *gsdf2* expression, protein localization, and tissue distribution throughout the life cycle, revealed a clear involvement of both paralogues in reproduction, with distinct roles in sex-specific processes. In a previous study from our group, we first identified two *gsdf* copies in this species and noted differential regulation in early maturing males, *gsdf1* was strongly downregulated, serving as a marker of precocious puberty, whereas *gsdf2* expression remained unchanged (18). Building on these initial insights, the present study further delineated the distinct functions of Gsdf paralogues during ontogenetic development. Both *gsdf1* and *gsdf2* were gonad-enriched genes, showing strong expression in gonads and only weak expression elsewhere. While our previous work suggested a nearly ubiquitous distribution (18), we have now clarified that *gsdf1* exhibits broader tissue distribution in adult of both sexes, with a clear dominant expression in gonads. This aligns with findings in most teleost species, where *gsdf* is typically gonad-specific (22, 70, 74, 115), or minimally expressed outside gonads (65, 116), reinforcing its classification as a local gonadal factor.

Consistently with other teleosts (7), in European sea bass, both *gsdf1* and *gsdf2* were expressed from early gonadal differentiation stages (150 dph) onward. At this time, sea bass females had already begun ovarian differentiation, evident by the formation of the ovarian cavity, while males remained histologically undifferentiated (53). This difference likely underlies the higher expression levels of *gsdf1* in females at 150 dph, compared to males. However, expression at this stage was still low compared to the pronounced increase at 200–250 dph coinciding with the completion of gonad differentiation, when ovaries are arranged in lamellae with previtellogenic oocytes and testes arranged in cysts with spermatogonia (53). Notably, *gsdf2* showed similar expression levels in both sexes across development, suggesting a sex-independent, secondary role in gonadal differentiation. Conversely, *gsdf1* expression was significantly higher and sex-biased, emphasizing its crucial role in testis differentiation. Our findings align with studies showing *gsdf* involvement before morphological sex differences arise, typically with male-biased expression associated with testis formation in both gonochoristic (7, 27, 117–119) and hermaphroditic species (10, 13, 14, 36). Immunolocalization confirmed the presence of Gsdf proteins in early gonadal development of European sea bass male and female juveniles, localized in both somatic cells surrounding germ cells and in the germ cells. These findings match with observations reported in other teleosts (22, 70, 115), suggesting that Gsdfs may be involved in the proliferation of SgA and oogonia in juvenile sea bass, with autocrine and/or paracrine action.

Puberty, marking first sexual maturity, occurs around 2 years of age in European sea bass males and 3 years in females, though precocious puberty can occur in cultured males within the first year of life (120). Our previous study in 9-month-old (September) European sea bass males suggested an involvement of *gsdf1* in puberty onset (18). The present time-course analysis highlighted temporal dynamics that were not captured in that single-point study, showing that both *gsdf1* and *gsdf2* were overall higher expressed in immature males compared to precocious ones, indicating downregulation associated with early maturation. This is in accordance with reports in yellowfin seabream (*Acanthopagrus latus*) (13) and Atlantic salmon (116), where *gsdf* is downregulated at the onset of puberty, consistent with a reduced requirement once spermatogenesis begins. Interestingly, in sea bass, the difference in *gsdf2* expression between immature and precocious testis was greater than that of *gsdf1*, which remained relatively stable up to November. This pattern may indicate a prolonged role for *gsdf1* and a more transient role for *gsdf2*, possibly restricted to spermatogonial proliferation in immature testis. These results are consistent with our previous study, in which *gsdf1* appeared downregulated in precocious males and *gsdf2* unchanged in a single September sampling (18), with apparent discrepancies explained by the improved temporal resolution in the current analysis.

Once puberty is attained, European sea bass reproduce multiple times over their lifespan, undergoing annual cycles through defined maturation phases (58–60), with spawning between January and March at our latitude (38). The present study revealed that *gsdf1* and *gsdf2* expression matched the detection of their encoded proteins in extracts of testis, ovary and follicular cells, indicating a strong mRNA-protein correspondence and supporting a gonad-specific function. Gene expression of both paralogues peaked in pre-meiotic (stage I) testes and pre-vitellogenic ovaries, then declined during the testicular meiotic phase and vitellogenesis, in parallel with hormonal changes, including rising levels of follicle-stimulating hormone (Fsh) (121), 11-ketotestosterone in males and oestradiol in females (63). This dynamic agrees with patterns seen in Atlantic salmon and spotted scat (74, 116), in which *gsdf* was upregulated during early gonadal differentiation and downregulated over the course of gametogenesis.

As previously observed in juveniles, adult males consistently showed higher *gsdf1* expression than *gsdf2*, indicating a more prominent role in testis physiology. Both paralogues peaked during spermatogonial mitotic proliferation, with Gsdf proteins localized in Sertoli cells surrounding SgA, as described in other species (10, 12, 16). Notably, the signal was spatially restricted within the testis, confined in Sertoli cells located in the central region of the lobules. A comparable localization pattern was observed in the protandrous yellowfin seabream, where *gsdf* mRNA was localized in Sertoli cell cytoplasm in the center of spermatogonial lobules (13). In adult Nile tilapia (30), medaka (9, 21), and threespot wrasse (*Halichoeres trimaculatus*) (36), *gsdf* mRNA was detected in the epithelial cells of the intratesticular efferent ducts, in addition to Sertoli cells surrounding SgA. This expression in ductal cells suggests that Gsdf in some species may acquire additional functions in the differentiation of male structure aside from gamete development (7). In seasonal breeders like European sea bass, the wall of the testicular lumen, composed mainly of Sertoli cells, experiences different structural and functional changes, such as the dedifferentiation of the Sertoli cells, or the remodeling of the testicular epithelium (122, 123). Our findings suggest that Gsdfs may be involved in these processes, which are regulated by hormonal and cellular factors.

Despite the male-biased expression of *gsdf*, analysis in female European sea bass ovaries provided intriguing differences between the two paralogs. Expression of *gsdf1* and *gsdf2* followed nearly identical trends, increasing during the post-spawning period and peaking at the onset of pre-vitellogenesis, coinciding with active ovarian remodeling, with the formation of new germ cells and follicles. These findings suggest that Gsdf could be involved in the ovarian reorganization and follicle development, as also supported by the immunodetection in follicular cells surrounding primary oocytes. Strikingly, expression profiles in isolated follicular cells unveiled *gsdf2* transcript levels nearly ten-fold higher than *gsdf1*, a difference masked in RNAs from whole-ovary due to germ cell contribution. Unlike its conserved role in testis development, *gsdf* function in female gonads appears more variable among teleosts, reflecting a higher degree of evolutionary plasticity. Gsdf-deficient medaka (37), zebrafish (28) and Nile tilapia (15) developed hyperplastic, sterile ovaries, with numerous immature follicles. In some teleost adult specimens, Gsdf localizes to somatic cells surrounding oogonia (12, 74), and more prominently in pre-vitellogenic follicles (13, 30, 124), consistent with our observations. In other species, the signal was detected at all developmental stages (116, 125) or in the germ cells (70). These findings indicate that, in a species-dependent manner, Gsdf may regulate ovarian development, fertility, and folliculogenesis by supporting and nourishing germ cells through autocrine or paracrine mechanisms.

A sex-specific pattern of Gsdf in European sea bass was further supported by protein detection in extracts of testis, ovary, and follicular cells. A band at approximately 25 kDa, consistent with the predicted molecular weight of the mature protein (23.384 kDa), was detected in gonadal extracts from both sexes (**Figure 8**) and in concentrated culture media from CHO cells expressing recombinant sea bass Gsdf1 or Gsdf2 (**Supplementary Figure 6**). However, marked differences were observed in natural tissues. In testis extracts, the ~25 kDa band was predominant, whereas ovarian tissue and isolated follicular cell extracts showed a dominant band at approximately 50–55 kDa, with the ~25 kDa signal remaining detectable but at lower intensity. As the antibody used did not discriminate between Gsdf1 and Gsdf2, both proteins were detected. These sex-dependent differences in apparent molecular weight are likely explained by distinct post-translational modifications; however, we demonstrated that the observed shift was not due to glycosylation (**Supplementary Figure 6**). Differences in cysteine content and distribution between Gsdf1 and Gsdf2 may contribute to the observed differences in apparent molecular weight, potentially affecting protein stability, folding properties, or susceptibility to post-translational modifications, and thereby influencing electrophoretic mobility. Accordingly, the higher molecular weight band observed in ovarian and follicular cell extracts may predominantly correspond to modified Gsdf2, as its coding gene is mainly expressed, whereas the ~25 kDa band detected in testis extracts likely reflects mainly Gsdf1, supporting a sex-dependent subfunctionalization of Gsdf in European sea bass. This interpretation is supported by similar observations in spotted scat, where Jiang et al. (74) reported a strong ~25 kDa Gsdf band in testis extracts and a weaker ~25 kDa signal together with an additional faint ~50 kDa band in ovarian tissue.

## 5 Conclusions

In the present study, phylogenetic reconstruction and synteny analyses unveiled that *gsdf* paralogues originated from independent duplication events in multiple fish lineages, rather than from the teleost-specific whole genome duplication (3R). Moreover, Gsdf is not exclusive to teleosts, but has evolutionary roots tracing back to the gnathostome ancestor. Despite this structural complexity, the conservation of surrounding syntenic blocks and promoter regulatory elements across species, strongly suggests functional constraints and a conserved role in early gonadal differentiation. In European sea bass, *gsdf1* and *gsdf2* paralogues were examined throughout the life cycle. Both were consistently associated with reproduction, acting during gonadal development before the appearance of phenotypic sex traits, negatively correlated with the onset of precocious puberty in males, and involved in the reproductive cycle of both sexes. Notably, the gonadal expression patterns in adults were sexually dimorphic: *gsdf1* was predominantly expressed in males, whereas *gsdf2* showed higher expression in females, particularly in follicular cells. These patterns suggest a sex-biased partitioning of ancestral *gsdf* functions in European sea bass, likely representing a subfunctionalization aligned with male and female reproductive roles. Protein localization analyses revealed Gsdf presence mainly in Sertoli cells surrounding type A spermatogonia in pre-meiotic testes and in follicular cells encompassing primary oocytes in pre-vitellogenic ovaries, consistent with a local paracrine regulation of early gametogenesis. Overall, our findings provide new insights into the evolutionary plasticity of gonad-related genes in teleosts, and highlight the contribution of lineage-specific duplications to the diversification of reproductive gene networks.

## Data Availability

The raw data supporting the conclusions of this article will be made available by the authors, without undue reservation.
