# Supplementary material for: Sex- and stage-dependent expression of gonadal soma-derived factor paralogues reveals functional and evolutionary divergence in European sea bass (Dicentrarchus labrax)
Source: Front Endocrinol (Lausanne). 2026 Mar 2;17:1779674. doi: 10.3389/fendo.2026.1779674 (PMC12989411; doi:10.3389/fendo.2026.1779674)
Supplement: Supplementary file 1 [file DataSheet1.pdf]

Supplementary Material

1 Supplementary Material and Methods

1.1 Recombinant sea bass Gsdf production

The complete ORF sequences of European sea bass *gsdf1* and *gsdf2* were cloned into the pcDNA3 vector, generating pcDNA3-*gsdf1* and pcDNA3-*gsdf2* expression plasmids. Chinese hamster ovary (CHO) cells were cultured in Dulbecco modified Eagle medium (DMEM) GlutaMAX (Life Technologies, Inc., Life Technologies™ Ltd., Paisley, Scotland, UK) supplemented with 5% Foetal Bovine Serum (FBS), and 100 U/mL Penicillin/Streptomycin (Pen/Strep, Life Technologies, Inc.), at 37 ºC in a humidified 5% CO_2 incubator. Cells were seeded in 6-well plates (~0.6 × 10^6 cells per well), grown to 75–80% confluence and transiently co-transfected with 2.5 µg of total DNA using Lipofectamine 3000 Reagent (Invitrogen) according to the manufacturer's protocol (final volume of transfection 250 µL/well). Plasmid DNA proportions in the transfections were as follows: 94% pcDNA3-*gsdf1* or pcDNA3-*gsdf2* and 6% pEGFP-N3 plasmid (Clontech; Palo Alto, CA, USA), constitutively expressing EGFP, used to check transfection efficiency. The day after transfections, medium was replaced, and cells were kept in a final volume of 2 ml in 6-well plates, up to 90-100% confluence. Then, cells were maintained at 25 ºC in a humidified 5% CO_2 incubator during 48 h, in order to allow production and secretion of recombinant proteins into the culture medium. The harvested media (CHO-Gsdf1 and CHO-Gsdf2) were centrifuged at 4 °C, 10000 × g for 10 min to remove dead cells debris and then the supernatants were concentrated by ultrafiltration at 4 °C, 3500 × g, for variable time, using Amicon Ultra-0.5 Centrifugal Filter (Merk Millipore Ltd.), cutoff of 10 kDa, following the manufacturer's protocol. SDS-PAGE followed by Western Blot analysis were performed to confirm the successful recombinant protein production.

1.2 Detection of Gsdfs by Western Blot

Total protein extraction from testis and ovary tissues was performed as illustrated in the paragraph 2.5 (Material and Methods section) of the main text. The potential glycosylation status of endogenous Gsdf proteins was checked by deglycosylation treatments.
A total of 35 µg of testis or ovary protein extracts were mixed with nuclease-free water to a final volume of 12 µl. Then, 1 µl of 5 % sodium dodecyl sulphate (SDS) and 1 µl of 1 M dithiothreitol (DTT) were added and samples were denatured by heating at 94 °C for 5 minutes. After cooling to room temperature for 5 minutes, 2 µl of 0.5 M sodium phosphate buffer (pH 7.5), 2 µl of 10% Nonidet P-40, and 2 µl (10 u) of recombinant peptide-N-glycosidase F (PNGase F; Promega) were added. For control samples (70 µg), water was used in place of PNGase F to ensure that any possible change in protein size could be exclusively attributed to deglycosylation process. The final reaction mixture was incubated at 37°C for 3 hours. Treated and not-treated extracts were subjected to SDS-PAGE followed by Western blot analysis.
For immunoblotting, either the whole not-treated samples (70 µg) or only a portion of the deglycosylation reactions (control extract, 35 µg; treated extract, 35 µg) were loaded, after mixing with Laemmli sample buffer and distilled water. Proteins were denatured at 95 °C for 5 min and separated under reducing conditions by SDS-PAGE (12 %), following standard procedures. Western blot was performed as described in in the paragraph 2.5 (Material and Methods section) of the main text.

2 Supplementary Figures and Tables

2.1 Supplementary Figures

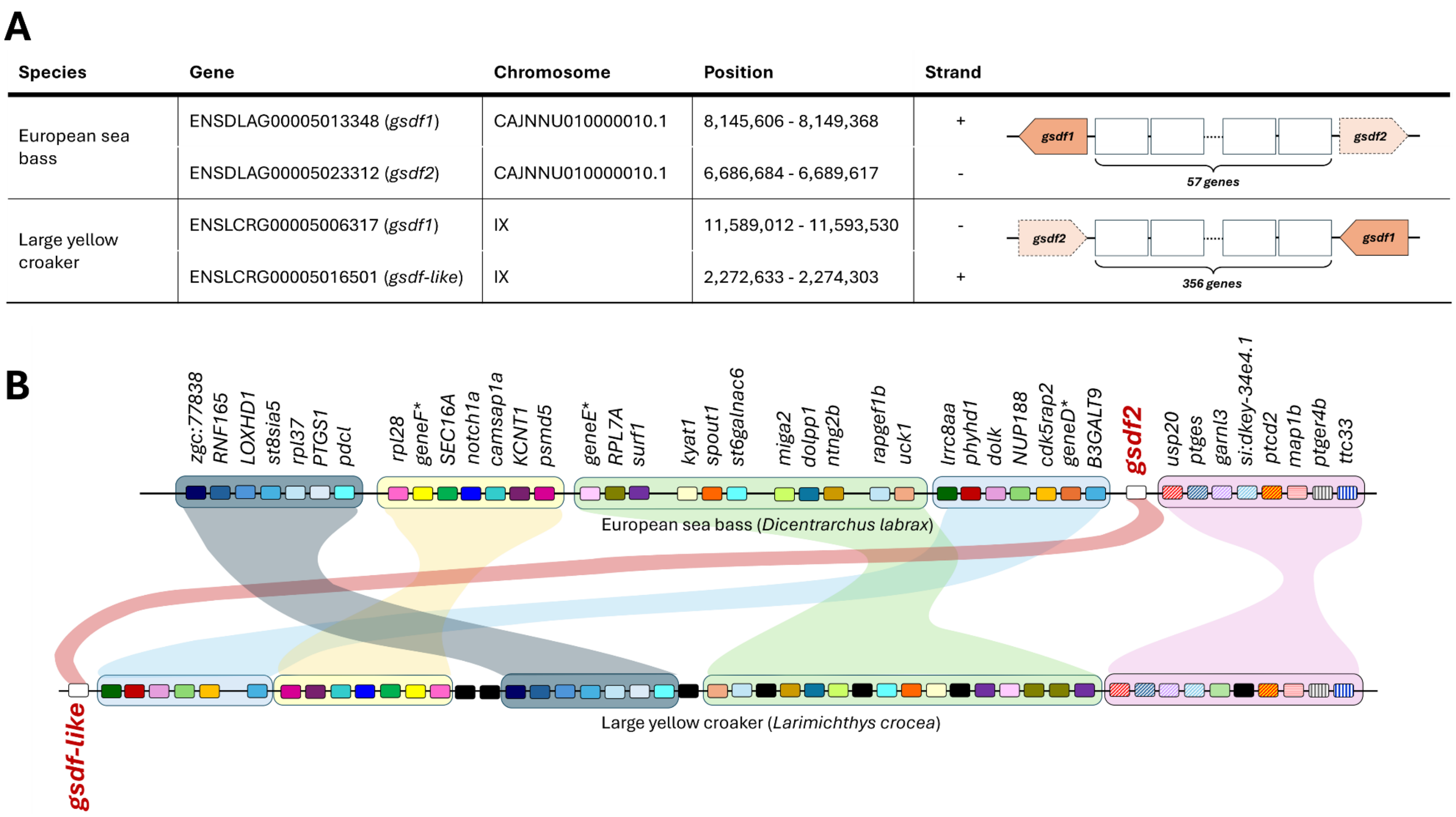

Species	Gene	Chromosome	Position	Strand
European sea bass	ENSDLAG00005013348 (*gsdf1*)	CAJNNU010000010.1	8,145,606 - 8,149,368	+
	ENSDLAG00005023312 (*gsdf2*)	CAJNNU010000010.1	6,686,684 - 6,689,617	-
Large yellow croaker	ENSLCRG00005006317 (*gsdf1*)	IX	11,589,012 - 11,593,530	-
	ENSLCRG00005016501 (*gsdf-like*)	IX	2,272,633 - 2,274,303	+

Supplementary Figure 1. (**A**) Summary table which describes *gsdf* duplicates in each species, indicating their chromosomal positions and illustrating their reciprocal localization. (**B**) Syntenic alignment of the chromosomic regions around European sea bass *gsdf2* and large yellow croaker *gsdf-like* genes. The synteny was analysed with Genomicus v110.01 using the European sea bass genes as reference. Chromosome segments are represented by black lines, coloured rectangles denote individual genes, with orthologs indicated by identical colours. Black squared represent non-orthologue genes, indicating a lack of synteny. Coloured rectangles indicated small conserved syntenic blocks that are rearranged in different configurations in the two species. Gene were retrieved from Ensembl and the abbreviation are listed in Supplementary Table 3.

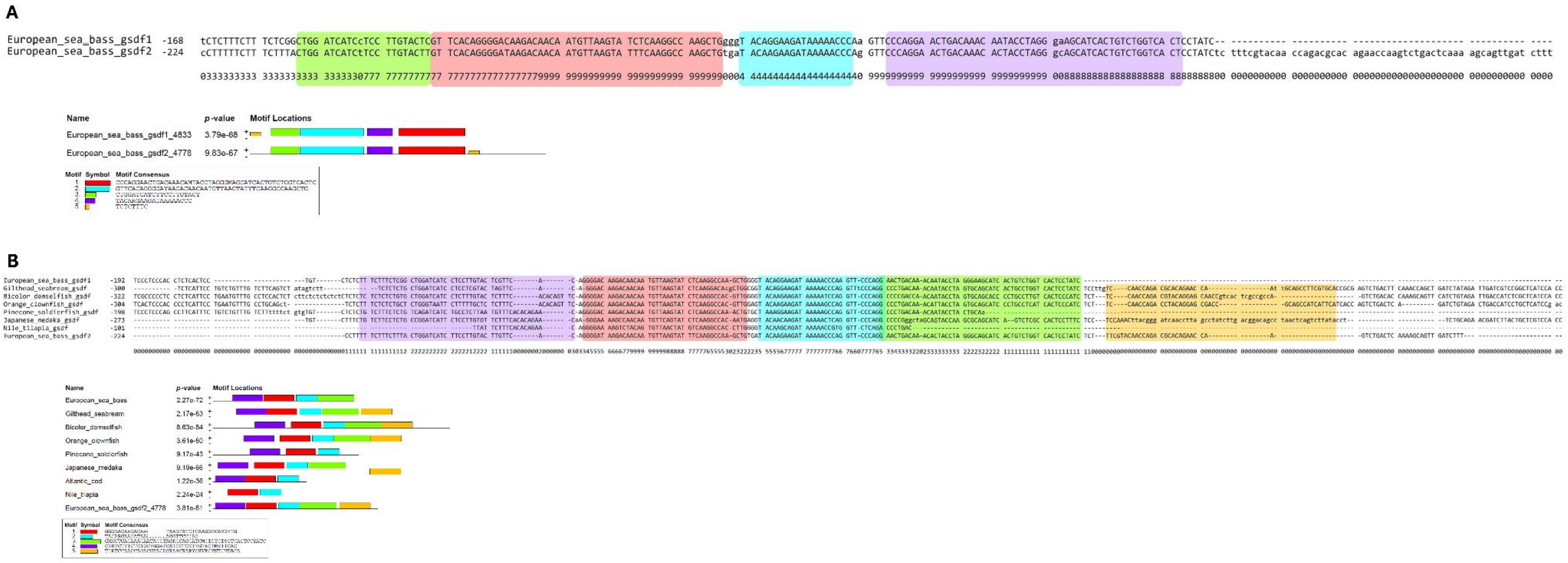

Supplementary Figure 2. Alignment of the 5 kb upstream regulatory regions of *gsdf1* and *gsdf2* genes from European sea bass (**A**), *gsdf* genes across teleost species. (**B**) Colored rectangles indicate predicted regulatory motifs. Pairwise alignment was performed by using the CHAOS/DIALIGN algorithm and alignment scores are shown as numerical values below the alignment blocks. Regulatory motifs were predicted by MEME Suite. Species used for the analysis: European sea bass (*Dicentrarchus labrax*), gilthead seabream (*Sparus aurata*), bicolor damselfish (*Stegastes partitus*), orange clownfish (*Amphiprion percula*), pinecone soldierfish (*Myripristis murdjan*), Japanese medaka (*Oryzias latipes*), Nile tilapia (*Oreochromis niloticus*).

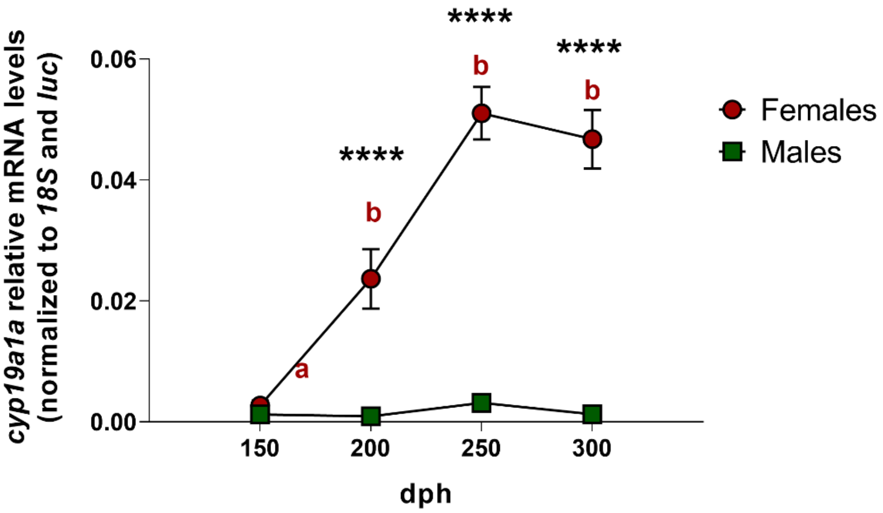

Supplementary Figure 3. Gonadal expression of *cyp19a1a* gene in European sea bass juveniles of both sexes during development from 150 dph to 300 dph (N=62). The expression was evaluated to perform sex assessment of all specimens. Data are reported as mean ± SEM and analysed by one-way ANOVA followed by post-hoc Tukey's test within each group, by Student's t-test between groups at each time point. Letters represent statistical significance ($p < 0.05$) among time points within an experimental group, asterisks indicated statistical difference (**** $p< 0.001$) between groups at each time point.

A

Month	Males							
	Total length (cm ± sd)	Total weight (kg ± sd)	I	II	III	IV	V	VI
May	53,42 ± 4,41	2,08 ± 0,57	16,67	0	0	0	0	83,33
Jun	46,58 ± 2,06	1,32 ± 0,15	100	0	0	0	0	0
Jul	49,33 ± 2,48	1,69 ± 0,28	100	0	0	0	0	0
Aug	51,50 ± 2,81	1,94 ± 0,31	100	0	0	0	0	0
Sep I	49,42 ± 3,44	1,79 ± 0,38	100	0	0	0	0	0
Sep II	49,67 ± 2,42	1,73 ± 0,35	66,67	33,33	0	0	0	0
Oct	50,67 ± 1,97	2,06 ± 0,25	33,33	66,67	0	0	0	0
Nov I	50,50 ± 1,77	2,02 ± 0,18	0	0	60,00	40,00	0	0
Nov II	56,50 ± 5,92	2,70 ± 0,44	0	0	16,67	83,33	0	0
Dec	50,33 ± 2,94	1,90 ± 0,36	0	0	0	50,00	50,00	0
Jan	48,92 ± 2,78	1,76 ± 0,3	0	0	0	66,67	33,33	0
Feb	57,33 ± 8,07	2,59 ± 0,38	0	0	0	16,67	83,33	0
Mar	51,50 ± 1,87	2,13 ± 0,17	0	0	0	0	100	0
Apr	51,00 ± 4,00	2,04 ± 0,60	0	0	0	0	33,33	67

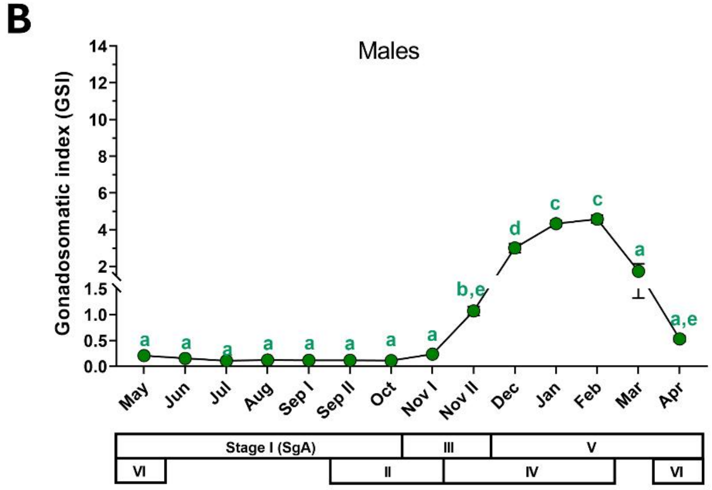

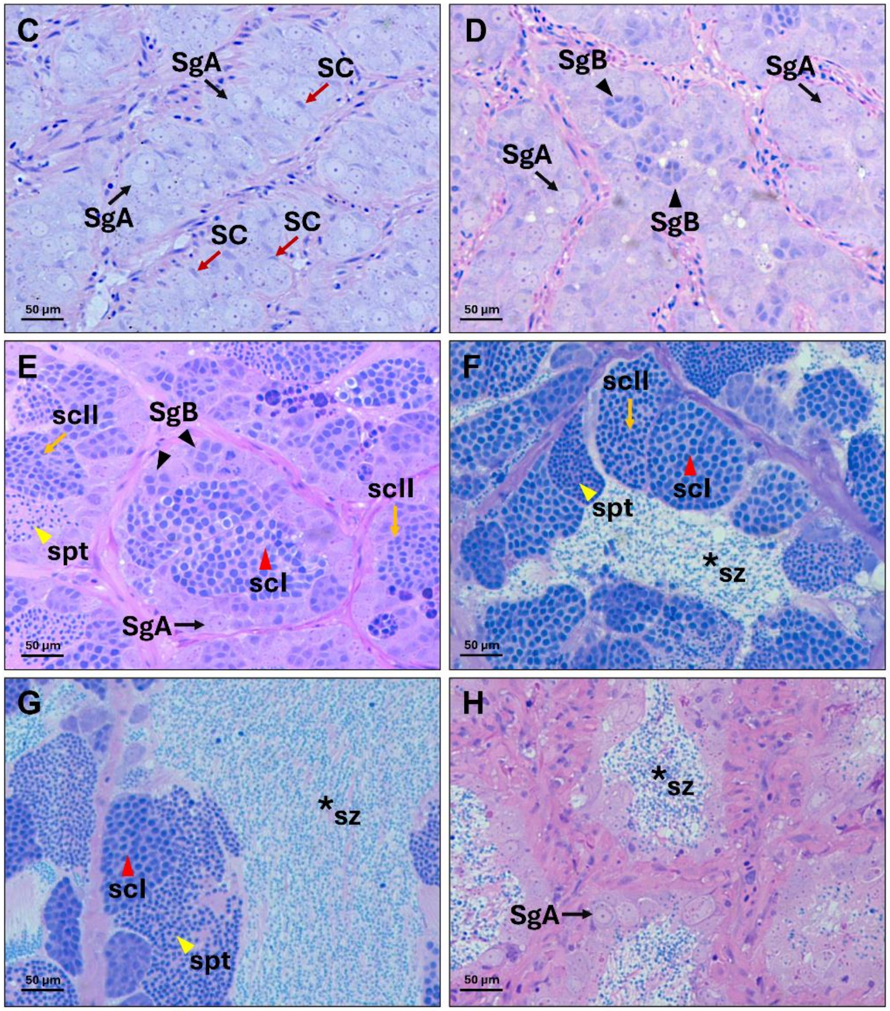

Supplementary Figure 4. Annual reproductive cycle in adult males of European sea bass: monthly biometric parameters, gonadosomatic index and histological panel. (**A**) Summary table showing mean total length and total weight of adult specimens and percentage distribution

of gonadal maturity stages, classified according to histological criteria, throughout the reproductive cycle. (**B**) Temporal changes in gonadosomatic index (GSI) during the annual reproductive season (N=6 fish/month). Data are reported as mean ± standard error of mean (SEM) and analysed by Kruskal-Wallis test, followed by post-hoc Dunn's test. Letters represent statistical significance ($p < 0.05$) among time points. (**C-H**) Representative histological sections for each of the six stages of sea bass testicular development. (**C**) Stage I, immature; (**D**) stage II, early recrudescence; (**E**) stage III, mid recrudescence; (**F**) stage IV, late recrudescence; (**G**) stage V, full spermiating testis; (**H**) stage VI, post-spawning. SgA, type A spermatogonia (black arrows); SC, Sertoli cells (red arrows); SgB, type B spermatogonia (black arrowheads); scI, primary spermatocytes (red arrowheads); scII, secondary spermatocytes (yellow arrows); spt, spermatids (yellow arrowheads); sz, spermatozoa (asterisks). Bars, 50 µm.

A

Month	Females							
	Total length (cm ± sd)	Total weight (kg ± sd)	Pre-vit	Early vit	Late vit	Post-vit	Matur/Ovul	Atresia
May	47,70 ± 9,18	1,68 ± 0,91	0	0	0	0	0	100
Jun	49,50 ± 1,73	1,52 ± 0,27	100	0	0	0	0	0
Jul	49,60 ± 1,52	1,73 ± 0,28	100	0	0	0	0	0
Aug	56,33 ± 3,06	2,48 ± 0,38	100	0	0	0	0	0
Sep I	55,62 ± 0,75	2,36 ± 0,16	100	0	0	0	0	0
Sep II	54,67 ± 2,31	2,46 ± 0,38	66,67	33,33	0	0	0	0
Oct	53,80 ± 0,45	2,45 ± 0,06	20,00	80,00	0	0	0	0
Nov I	56,50 ± 2,36	2,68 ± 0,28	20,00	20,00	60,00	0	0	0
Nov II	53,00 ± 3,08	2,53 ± 0,48	0	0	80,00	20,00	0	0
Dec	61,40 ± 8,96	2,95 ± 0,62	0	0	20,00	80,00	0	0
Jan	55,80 ± 2,95	3,14 ± 0,46	0	0	0	20,00	80,00	0
Feb	54,80 ± 8,23	2,68 ± 0,79	0	0	0	20,00	80,00	0
Mar	59,00 ± 0,71	3,14 ± 0,26	0	0	0	0	60,00	40,00
Apr	60,80 ± 4,71	2,86 ± 0,23	0	0	0	0	0	100

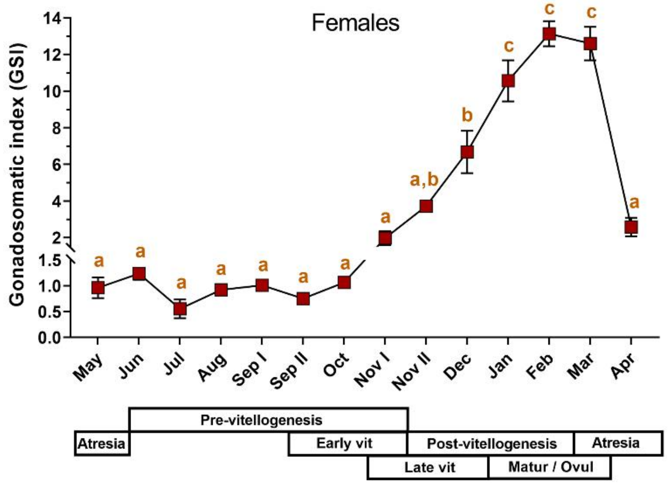

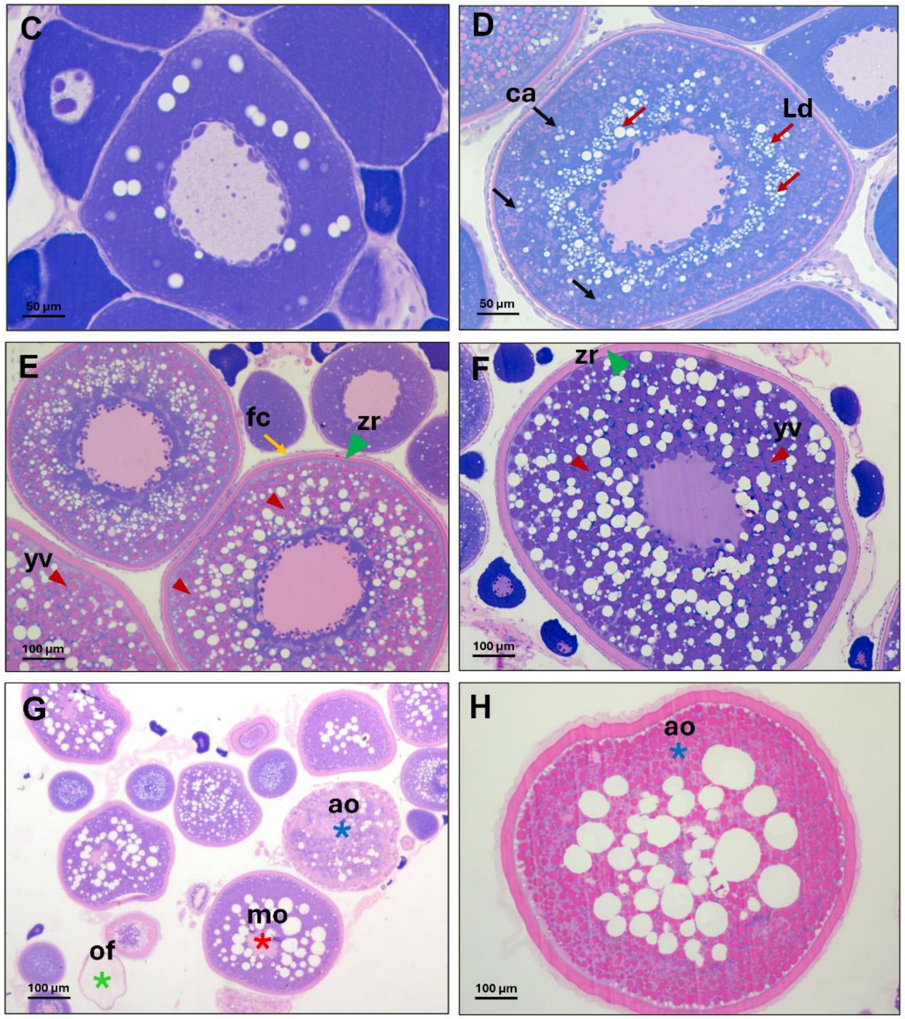

Supplementary Figure 5. Annual reproductive cycle in adult females of European sea bass: monthly biometric parameters, gonadosomatic index and histological panel. (**A**) Summary table showing mean total length and total weight of adult specimens and percentage distribution

of gonadal maturity stages, classified according to histological criteria, throughout the reproductive cycle. (**B**) Temporal changes in gonadosomatic index (GSI) during the annual reproductive season (N=5 fish/month). Data are reported as mean ± standard error of mean (SEM) and analysed by Kruskal-Wallis test, followed by post-hoc Dunn's test. Letters represent statistical significance ($p < 0.05$) among time points. (**C-H**) Representative histological sections for each of the six stages of sea bass ovarian development showing seasonal changes in cell components. (**C**) Pre-vitellogenesis; (**D**) early vitellogenesis; (**E**) late vitellogenesis; (**F**) post-vitellogenesis; (**G**) maturation/ovulation; (**H**) atresia. ca, cortical alveoli (black arrows); Ld, lipid droplets (red arrows); fc, follicular cells (yellow arrows); yv, yolk vesicle (red arrowheads); zr, zona radiata (green arrowheads); ao, atretic oocytes (blue asterisks); mo, maturing oocytes (red asterisk); of, ovulated follicle (green asterisk). Bars, 50 µm (C-D), 100 µm (E-H).

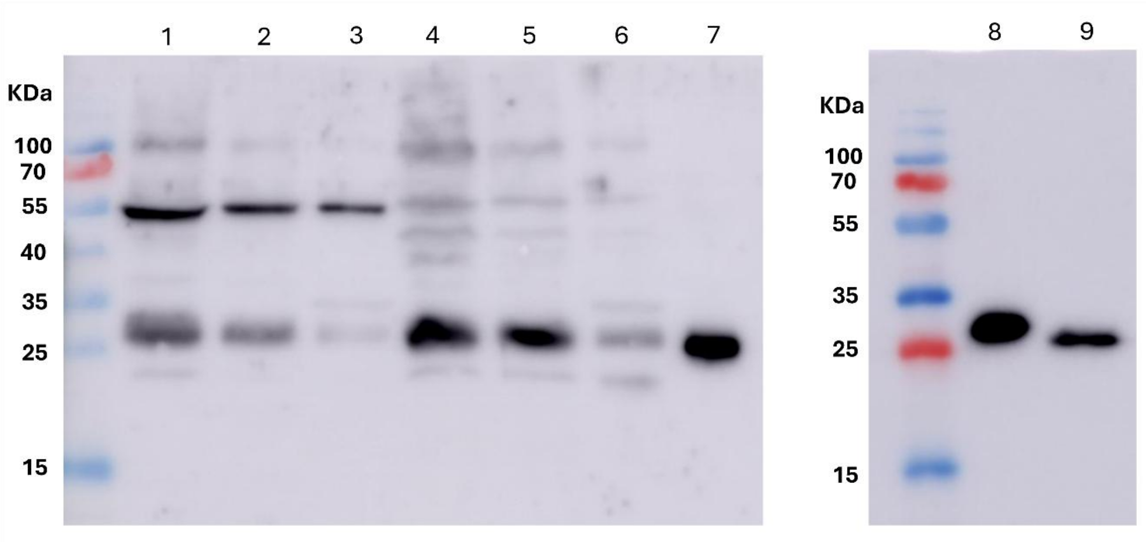

Supplementary Figure 6. Validation of the specific antibody against European sea bass Gsdf proteins by Western blot. Gsdf proteins were detected under reducing conditions. Samples are the following: ovarian extracts (70 µg, lane 1) and ovarian extracts incubated at 37 ºC for 3 h without (35 µg, lane 2) or with (9 µg, lane 3) PNGase F. Testis extracts (70 µg, lanes 4) and testis extracts incubated at 37 ºC for 3 h without (35 µg, lane 2) or with (9 µg, lane 3) PNGase F. Concentrated culture media of CHO cells expressing recombinant sea bass Gsdf1 (lanes 7-8) or Gsdf2 (lane 9).

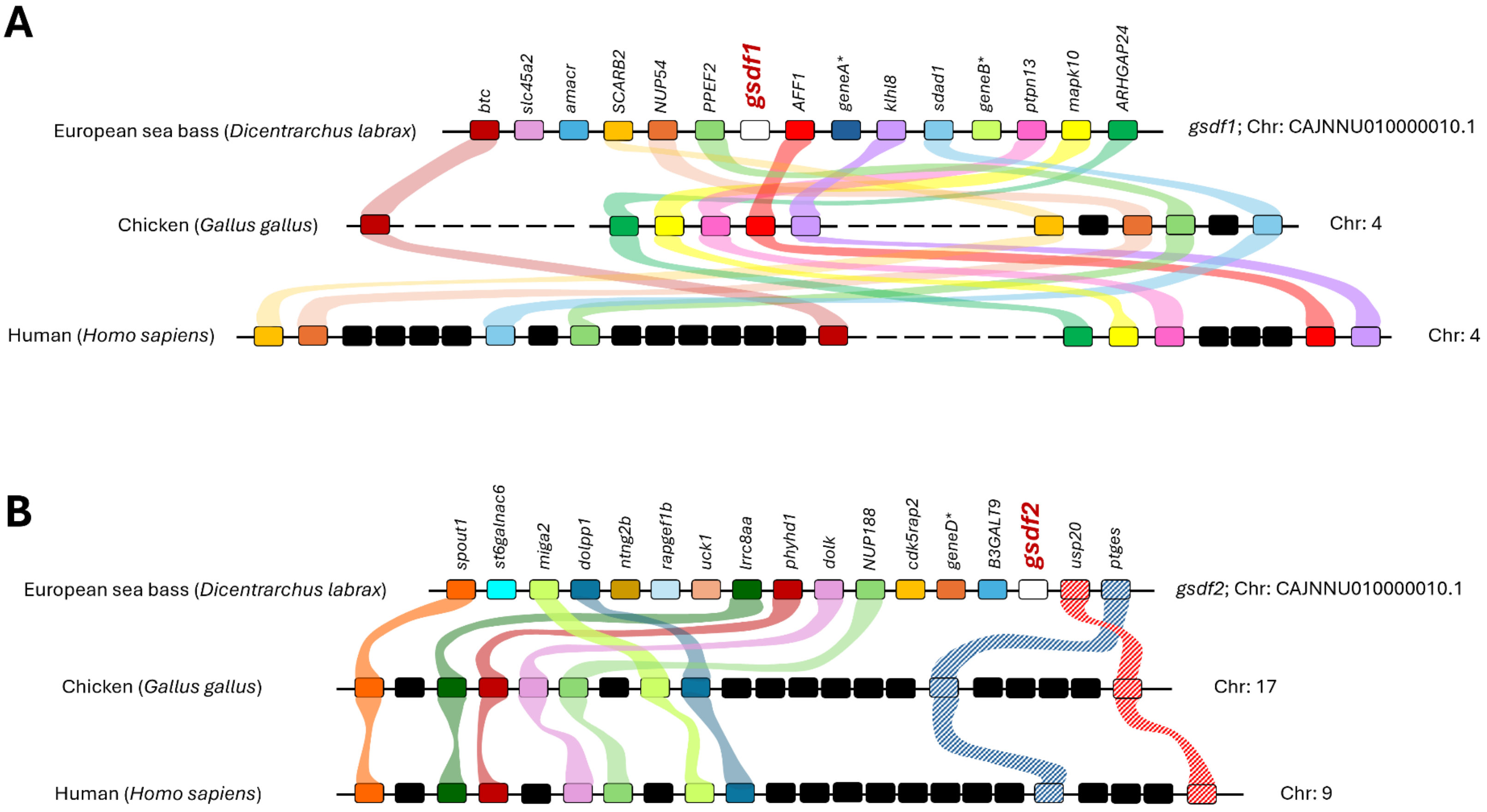

Supplementary Figure 7. Syntenic alignment of the conserved flanking genes surrounding European sea bass *gsdf1* (**A**) or *gsdf2* (**B**) in chicken and human. The synteny was analysed with Genomicus v110.01 using the European sea bass genes as reference. Chromosome segments are represented by black lines, and dashed line indicate the extension of the same chromosome until reaching other loci of interest. Coloured rectangles denote individual genes, with orthologs indicated by identical colours. Black squared represent non-orthologue genes, indicating a lack of synteny. Gene were retrieved from Ensembl and the abbreviation are listed in Supplementary Table 3.

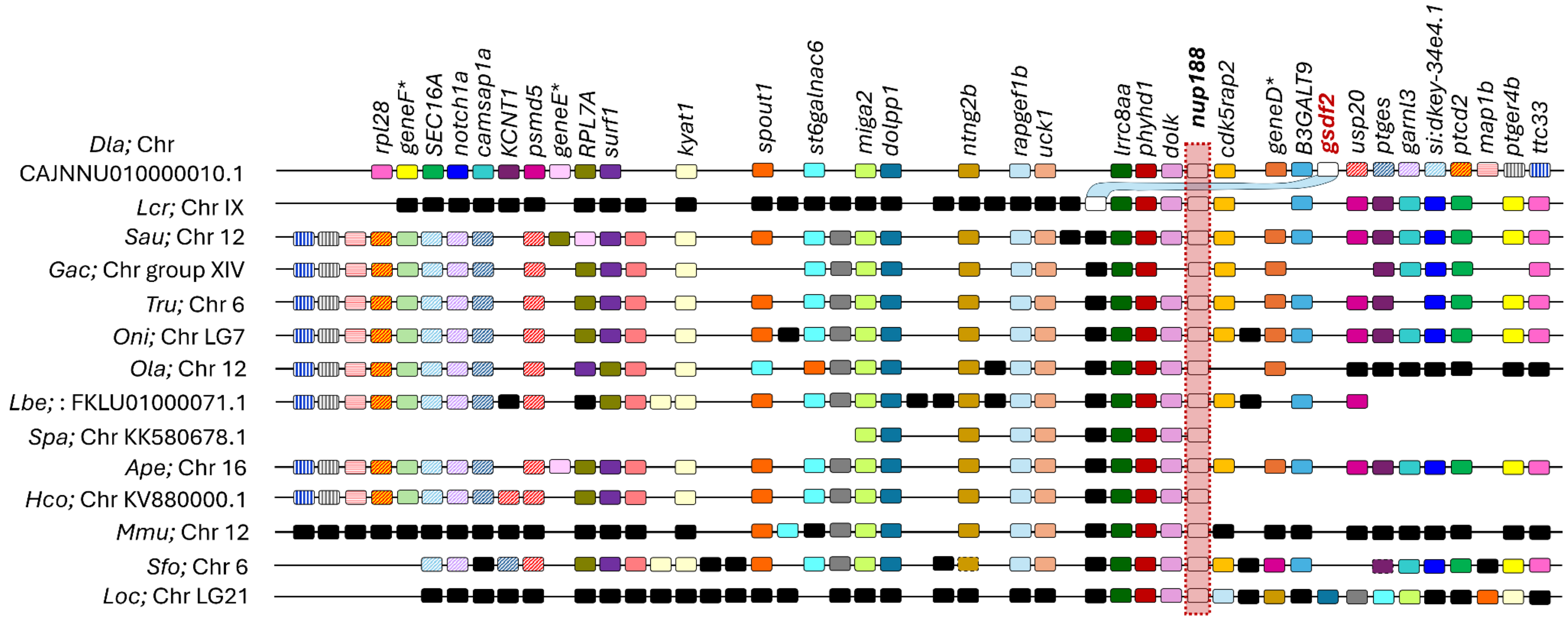

Supplementary Figure 8. Syntenic alignment of the chromosomic regions around European sea bass *gsdf2* in different teleost species. The synteny was analysed with Genomicus v110.01 using the European sea bass *nup188* gene as reference. Chromosome segments are represented by black lines, coloured rectangles denote individual genes, with orthologs indicated by identical colours. Paralogues are represented by same colour but dashed border. The red vertical square highlights *gsdf* orthologues in different species. A black line between two genes is equivalent to a break in the continuity of the alignment. Black squared represent non-orthologue genes, indicating a lack of synteny. Genes in the flanking regions were retrieved from Ensembl and the abbreviation are listed in Supplementary Table S3. *nup188* orthologues (listed in Supplementary Table S1) were extracted from Ensembl genome assemblies of European sea bass (*Dla*) (*Dicentrarchus labrax*), large yellow croaker (*Lcr*) (*Larimichthys crocea*), gilthead seabream (*Sau*) (*Sparus aurata*), stickleback (*Gac*) (*Gasterosteus aculeatus*), fugu (*Tru*) (*Takifugu rubripes*), Nile tilatia (*Oni*) (*Oreochromis niloticus*), Japanese medaka (*Ola*) (*Oryzias latipes*), ballan wrasse (*Lbe*) (*Labrus bergylta*), bicolor damselfish (*Spa*) (*Stegastes partitus*), orange clownfish (*Ape*) (*Amphiprion percula*), tiger tail seahorse (*Hco*) (*Hippocampus comes*), pinecone soldierfish (*Mmu*) (*Myripristis murdjan*), Asian bonytongue (*Sfo*) (*Scleropages formosus*), spotted gar (*Loc*) (*Lepisosteus oculatus*).

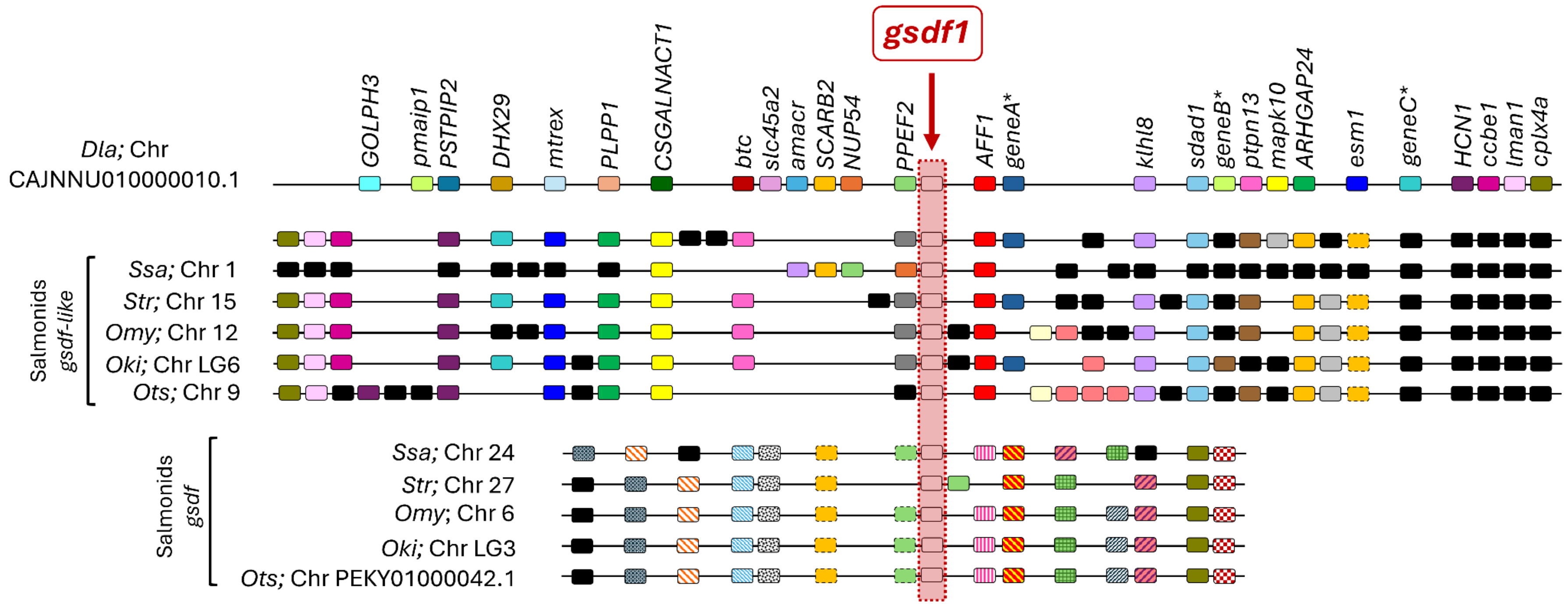

Supplementary Figure 9. Syntenic alignment of the chromosomic regions around European sea bass *gsdf1* in different salmonids species. The synteny was analysed with Genomicus v110.01 using the European sea bass *gsdf1* as reference. Chromosome segments are represented by black lines, coloured rectangles denote individual genes, with orthologs indicated by identical colours. Paralogues are represented by same colour but dashed border. The red vertical square highlights *gsdf* orthologues in different species. A black line between two genes is equivalent to a break in the continuity of the alignment. Black squared represent non-orthologous genes, indicating a lack of synteny. Genes in the flanking regions were retrieved from Ensembl and the abbreviation are listed in Supplementary Table S3. Gsdf orthologues (listed in Supplementary Table S1) were extracted from Ensembl genome assemblies of European sea bass (*Dla*) (*Dicentrarchus labrax*), Atlantic salmon (*Ssa*) (*Salmo salar*), river trout (*Str*) (*Salmo trutta*), rainbow trout (*Omy*) (*Oncorhynchus mykiss*), Coho salmon (*Oki*) (*Oncorhynchus mykiss*), chinook salmon (*Ots*) (*Oncorhynchus tshawytscha).*

2.2 Supplementary Tables

Supplementary Table 1. List of accession numbers used for the phylogenetic study of *gsdf*.

Name designed in this study	Ensembl/NCBI Accession number Gene	Ensembl/NCBI Accession number Protein	Animal	Species	Synteny Analysis	Putative promoter study
Gsdf1 European sea bass	ENSDLAG00005013348	ENSDLAP00005029669	European sea bass	*Dicentrarchus labrax*	X	X
Gsdf2 European sea bass	ENSDLAG00005023312	ENSDLAP00005054424	European sea bass	*Dicentrarchus labrax*	X	X
Gsdf Large yellow croaker	ENSLCRG00005006317	ENSLCRP00005014780	Large yellow croaker	*Larimichthys crocea*	X	X
Gsdf-like Large yellow croaker	ENSLCRG00005016501	ENSLCRP00005042944	Large yellow croaker	*Larimichthys crocea*	X	
Gsdf Gilthead seabream	ENSSAUG00010009864	ENSSAUP00010022368	Gilthead seabream	*Sparus aurata*	X	X
Gsdf Stickleback	ENSGACG00000016330	ENSGACP00000021554	Three-spined stickleback	*Gasterosteus aculeatus*	X	X
Gsdf Takifugu	ENSTRUG00000028164	ENSTRUP00000086647	Fugu	*Takifugu rubripes*	X	
Gsdf Nile tilapia	ENSONIG00000007633	ENSONIP00000009618	Nile tilapia	*Oreochromis niloticus*	X	X
Gsdf Japanese medaka	ENSORLG00000022492	ENSORLP00000042059	Japanese medaka	*Oryzias latipes*	X	X
Gsdf Asian bonytongue	ENSSFOG00015021728	ENSSFOP00015039701	Asian bonytongue	*Scleropages formosus*	X	
Gsdf Spotted gar	ENSLOCG00000004013	ENSLOCP00000004794	Spotted gar	*Lepisosteus oculatus*	X	
Gsdf P. kingsleyae	ENSPKIG00000024213	ENSPKIP00000008905	Paramormyrops kingsleyae	*Paramormyrops kingsleyae*		
Gsdf Ballan wrasse	ENSLBEG00000016971	ENSLBEP00000022096	Ballan wrasse	*Labrus bergylta*	X	X
Gsdf -like Ballan wrasse	ENSLBEG00000007423	ENSLBEP00000009593	Ballan wrasse	*Labrus bergylta*	X	
Gsdf Bicolor damselfish	ENSSPAG00000003999	ENSSPAP00000005158	Bicolor damselfish	*Stegastes partitus*	X	X
Gsdf -like Bicolor damselfish	ENSSPAG00000002047	ENSSPAP00000002661	Bicolor damselfish	*Stegastes partitus*	X	
Gsdf Orange clownfish	ENSAPEG00000018952	ENSAPEP00000026668	Orange clownfish	*Amphiprion percula*	X	X
Gsdf-like Orange clownfish	ENSAPEG00000018832	ENSAPEP00000026640	Orange clownfish	*Amphiprion percula*		
Gsdf Tiger tail seahorse	ENSHCOG00000020179	ENSHCOP00000016443	Tiger tail seahorse	*Hippocampus comes*	X	
Gsdf-like Tiger tail seahorse	ENSHCOG00000019945	ENSHCOP00000016186	Tiger tail seahorse	*Hippocampus comes*	X	
Gsdf Pinecone soldierfish	ENSMMDG00005002721	ENSMMDP00005004914	Pinecone soldierfish	*Myripristis murdjan*	X	X
Gsdf-like Pinecone soldierfish	ENSMMDG00005002729	ENSMMDP00005004923	Pinecone soldierfish	*Myripristis murdjan*		

Gsdf Barramundi perch	ENSLCAG00010004070	ENSLCAP00010008405	Barramundi perch	*Lates calcarifer*	
Gsdf Greater amberjack	ENSSDUG00000023776	ENSSDUP00000033102	Greater amberjack	*Seriola dumerili*	
Gsdf Lumpfish	ENSCLMG00005001380	ENSCLMP00005003112	Lumpfish	*Cyclopterus lumpus*	
Gsdf Chinook salmon	ENSOTSG00005004728	ENSOTSP00005008697	Chinook salmon	*Oncorhynchus tshawytscha*	
Gsdf-like Chinook salmon	ENSOTSG00005042956	ENSOTSP00005091701	Chinook salmon	*Oncorhynchus tshawytscha*	
Gsdf Coho salmon	ENSOKIG00005028175	ENSOKIP00005065613	Coho salmon	*Oncorhynchus kisutch*	
Gsdf-like Coho salmon	ENSOKIG00005019188	ENSOKIP00005045539	Coho salmon	*Oncorhynchus kisutch*	
Gsdf Rainbow trout	ENSOMYG00000015157	ENSOMYP00000032407	Rainbow trout	*Oncorhynchus mykiss*	X
Gsdf-like Rainbow trout	ENSOMYG00000002600	ENSOMYP00000004997	Rainbow trout	*Oncorhynchus mykiss*	X
Gsdf Atlantic salmon	ENSSSAG00000065603	ENSSSAP00000076514	Atlantic salmon	*Salmo salar*	
Gsdf-like Atlantic salmon	ENSSSAG00000070512	ENSSSAP00000154889	Atlantic salmon	*Salmo salar*	
Gsdf-like2 Atlantic salmon	ENSSSAG00000031281	ENSSSAP00000027013	Atlantic salmon	*Salmo salar*	
Gsdf River trout	ENSSTUG00000012514	ENSSTUP00000028909	River trout (Brown trout)	*Salmo trutta*	
Gsdf-like River trout	ENSSTUG00000009055	ENSSTUP00000020428	River trout (Brown trout)	*Salmo trutta*	
Gsdf Small-spotted catshark	XM_038787356.1	XP_038643284.1	Small-spotted catshark	*Scyliorhinus canicula*	
Gsdf Lesser devil ray	XM_063036145.1	XP_062892215.1.	Lesser devil ray	*Mobula hypostoma*	
Gsdf Australian ghostshark	XM_007911053	XP_007909244.1	Australian ghostshark	*Callorhinchus milii*	
Gsdf West Indian Ocean coelacanth	XM_064551916.1	XP_064407986.1	West Indian Ocean coelacanth	*Latimeria chalumnae*	
Gsdf Indonesian coelacanth	HF562312.1	CCP19133.1	Indonesian coelacanth	*Latimeria menadoensis*	
Gsdf African lungfish	MH329974.1 (complete cds)	AWT24641.1	African lungfish	*Protopterus annectens*	
Gsdf Chinese fire-bellied newt	MN923222.1 (complete cds)	QIS93397.1	Chinese fire-bellied newt	*Hypselotriton orientalis*	
Gsdf Unicolor caecilian	XM_030219967.1	XP_030075827.1	Unicolor caecilian	*Microcaecilia unicolor*	

Supplementary Table 2. List of genes in the flanking upstream and downstream regions of *gsdf1* and *gsdf2,* retrieved from Ensembl. Asterisks indicate *gsdf* homologs without assigned gene names.

Name designed in this study	Ensembl Accession number Gene	Animal	Species	Position	Figure
PPEF2	ENSDLAG00005012856	European sea bass	*Dicentrarchus labrax*	*gsdf1* upstream region	2A
NUP54	ENSDLAG00005012623	European sea bass	*Dicentrarchus labrax*	*gsdf1* upstream region	2A
SCARB2	ENSDLAG00005012454	European sea bass	*Dicentrarchus labrax*	*gsdf1* upstream region	2A
amacr	ENSDLAG00005033682	European sea bass	*Dicentrarchus labrax*	*gsdf1* upstream region	2A
slc45a2	ENSDLAG00005012438	European sea bass	*Dicentrarchus labrax*	*gsdf1* upstream region	2A
rxfp3	ENSLCRG00005006499	Large yellow croaker	*Larimichthys crocea*	*gsdf1* upstream region	2A
btc	ENSDLAG00005012399	European sea bass	*Dicentrarchus labrax*	*gsdf1* upstream region	2A
gng10	ENSLCRG00005006501	Large yellow croaker	*Larimichthys crocea*	*gsdf1* upstream region	2A
nxnl2	ENSLCRG00005006503	Large yellow croaker	*Larimichthys crocea*	*gsdf1* upstream region	2A
CSGALNACT1	ENSDLAG00005029474	European sea bass	*Dicentrarchus labrax*	*gsdf1* upstream region	2A
PLPP1	ENSDLAG00005033711	European sea bass	*Dicentrarchus labrax*	*gsdf1* upstream region	2A
mtrex	ENSDLAG00005011692	European sea bass	*Dicentrarchus labrax*	*gsdf1* upstream region	2A
DHX29	ENSDLAG00005010274	European sea bass	*Dicentrarchus labrax*	*gsdf1* upstream region	2A
PSTPIP2	ENSDLAG00005010246	European sea bass	*Dicentrarchus labrax*	*gsdf1* upstream region	2A
pmaip1	ENSDLAG00005033974	European sea bass	*Dicentrarchus labrax*	*gsdf1* upstream region	2A
GOLPH3	ENSDLAG00005010229	European sea bass	*Dicentrarchus labrax*	*gsdf1* upstream region	2A
ITGA1	ENSDLAG00005009932	European sea bass	*Dicentrarchus labrax*	*gsdf1* upstream region	2A
AFF1	ENSDLAG00005013358	European sea bass	*Dicentrarchus labrax*	*gsdf1* downstream region	2A
*geneA**	ENSDLAG00005013395	European sea bass	*Dicentrarchus labrax*	*gsdf1* downstream region	2A
klhl8	ENSDLAG00005013484	European sea bass	*Dicentrarchus labrax*	*gsdf1* downstream region	2A
sdad1	ENSDLAG00005013541	European sea bass	*Dicentrarchus labrax*	*gsdf1* downstream region	2A
*geneB**	ENSDLAG00005013613	European sea bass	*Dicentrarchus labrax*	*gsdf1* downstream region	2A
ptpn13	ENSDLAG00005013629	European sea bass	*Dicentrarchus labrax*	*gsdf1* downstream region	2A
mapk10	ENSDLAG00005013846	European sea bass	*Dicentrarchus labrax*	*gsdf1* downstream region	2A
ARHGAP24	ENSDLAG00005014135	European sea bass	*Dicentrarchus labrax*	*gsdf1* downstream region	2A
esm1	ENSDLAG00005034902	European sea bass	*Dicentrarchus labrax*	*gsdf1* downstream region	2A
*geneC**	ENSDLAG00005014272	European sea bass	*Dicentrarchus labrax*	*gsdf1* downstream region	2A

HCN1	ENSDLAG00005015918	European sea bass	*Dicentrarchus labrax*	*gsdf1* downstream region	2A
ccbe1	ENSDLAG00005015956	European sea bass	*Dicentrarchus labrax*	*gsdf1* downstream region	2A
lman1	ENSDLAG00005015980	European sea bass	*Dicentrarchus labrax*	*gsdf1* downstream region	2A
cplx4a	ENSDLAG00005016198	European sea bass	*Dicentrarchus labrax*	*gsdf1* downstream region	2A
B3GALT9	ENSDLAG00005023314	European sea bass	*Dicentrarchus labrax*	*gsdf2* upstream region	2B
*geneD**	ENSDLAG00005023321	European sea bass	*Dicentrarchus labrax*	*gsdf2* upstream region	2B
cdk5rap2	ENSDLAG00005023329	European sea bass	*Dicentrarchus labrax*	*gsdf2* upstream region	2B
NUP188	ENSDLAG00005023401	European sea bass	*Dicentrarchus labrax*	*gsdf2* upstream region	2B
dolk	ENSDLAG00005028297	European sea bass	*Dicentrarchus labrax*	*gsdf2* upstream region	2B
phyhd1	ENSDLAG00005023448	European sea bass	*Dicentrarchus labrax*	*gsdf2* upstream region	2B
lrrc8aa	ENSDLAG00005023479	European sea bass	*Dicentrarchus labrax*	*gsdf2* upstream region	2B
uck1	ENSDLAG00005023486	European sea bass	*Dicentrarchus labrax*	*gsdf2* upstream region	2B
rapgef1b	ENSDLAG00005023495	European sea bass	*Dicentrarchus labrax*	*gsdf2* upstream region	2B
ntng2b	ENSDLAG00005023653	European sea bass	*Dicentrarchus labrax*	*gsdf2* upstream region	2B
dolpp1	ENSDLAG00005023666	European sea bass	*Dicentrarchus labrax*	*gsdf2* upstream region	2B
miga2	ENSDLAG00005023677	European sea bass	*Dicentrarchus labrax*	*gsdf2* upstream region	2B
st6galnac6	ENSDLAG00005000515	European sea bass	*Dicentrarchus labrax*	*gsdf2* upstream region	2B
spout1	ENSDLAG00005000535	European sea bass	*Dicentrarchus labrax*	*gsdf2* upstream region	2B
usp20	ENSDLAG00005022729	European sea bass	*Dicentrarchus labrax*	*gsdf2* downstream region	2B
ptges	ENSDLAG00005033508	European sea bass	*Dicentrarchus labrax*	*gsdf2* downstream region	2B
garnl3	ENSDLAG00005022433	European sea bass	*Dicentrarchus labrax*	*gsdf2* downstream region	2B
si:dkey-34e4.1	ENSDLAG00005022405	European sea bass	*Dicentrarchus labrax*	*gsdf2* downstream region	2B
ptcd2	ENSDLAG00005029467	European sea bass	*Dicentrarchus labrax*	*gsdf2* downstream region	2B
map1b	ENSDLAG00005022393	European sea bass	*Dicentrarchus labrax*	*gsdf2* downstream region	2B
ptger4b	ENSDLAG00005028637	European sea bass	*Dicentrarchus labrax*	*gsdf2* downstream region	2B
ttc33	ENSDLAG00005022383	European sea bass	*Dicentrarchus labrax*	*gsdf2* downstream region	2B
prkaa1	ENSDLAG00005022332	European sea bass	*Dicentrarchus labrax*	*gsdf2* downstream region	2B
aif1l	ENSDLAG00005033318	European sea bass	*Dicentrarchus labrax*	*gsdf2* downstream region	2B
lamc3	ENSDLAG00005022319	European sea bass	*Dicentrarchus labrax*	*gsdf2* downstream region	2B
fibcd1b	ENSDLAG00005027095	European sea bass	*Dicentrarchus labrax*	*gsdf2* downstream region	2B
zgc:63972	ENSDLAG00005022299	European sea bass	*Dicentrarchus labrax*	*gsdf2* downstream region	2B

dab2ipb	ENSDLAG00005020932	European sea bass	*Dicentrarchus labrax*	*gsdf2* downstream region	2B
kyat1	ENSDLAG00005000610	European sea bass	*Dicentrarchus labrax*	*gsdf2* downstream region	Suppl. Fig. 8
surf1	ENSDLAG00005030041	European sea bass	*Dicentrarchus labrax*	*gsdf2* downstream region	Suppl. Fig. 8
RPL7A	ENSDLAG00005000683	European sea bass	*Dicentrarchus labrax*	*gsdf2* downstream region	Suppl. Fig. 8
*geneE**	ENSDLAG00005000680	European sea bass	*Dicentrarchus labrax*	*gsdf2* downstream region	Suppl. Fig. 8
psmd5	ENSDLAG00005000746	European sea bass	*Dicentrarchus labrax*	*gsdf2* downstream region	Suppl. Fig. 8
KCNT1	ENSDLAG00005001124	European sea bass	*Dicentrarchus labrax*	*gsdf2* downstream region	Suppl. Fig. 8
camsap1a	ENSDLAG00005001449	European sea bass	*Dicentrarchus labrax*	*gsdf2* downstream region	Suppl. Fig. 8
notch1a	ENSDLAG00005004755	European sea bass	*Dicentrarchus labrax*	*gsdf2* downstream region	Suppl. Fig. 8
SEC16A	ENSDLAG00005030236	European sea bass	*Dicentrarchus labrax*	*gsdf2* downstream region	Suppl. Fig. 8
*geneF**	ENSDLAG00005006652	European sea bass	*Dicentrarchus labrax*	*gsdf2* downstream region	Suppl. Fig. 8
rpl28	ENSDLAG00005006661	European sea bass	*Dicentrarchus labrax*	*gsdf2* downstream region	Suppl. Fig. 8

Supplementary Table 3. Contingency table indicating sequence identity percentages obtained by intra- and inter-specific comparison between Gsdf and Gsdf-like. European sea bass Gsdf1 and Gsdf2 were compared with all copies from different species. Coloured boxes indicated sequence identity between sea bass Gsdf1 and Gsdf from other species (green); sea bass Gsdf2 and Gsdf-like from other species (orange); Gsdf and Gsdf-like within each species (purple).

		European sea bass		Large yellow croaker	Ballan wrasse	Bicolor damselfish	Orange clownfish	Tiger tail seahorse	Pinecone soldierfish	Rainbow trout
		Gsdf1	Gsdf2	Gsdf	Gsdf	Gsdf	Gsdf	Gsdf	Gsdf	Gsdf
	Gsdf2	85.71%								
Large yellow croaker	Gsdf	69.59%	67.28%							
	Gsdf-like	70.70%	68.64%	62.91%						
Gilthead seabream	Gsdf	70.51%	69.59%							
Stickleback	Gsdf	68.60%	63.63%							
Fugu	Gsdf	58.53%	55.30%							
Nile tilapia	Gsdf	63.13%	61.29%							
Japanese medaka	Gsdf	60.98%	58.99%							
Ballan wrasse	Gsdf	66.06%	63.76%							
	Gsdf-like	63.01%	61.64%		65.74%					
Bicolor damselfish	Gsdf	67.44%	66.51%							
	Gsdf-like	64.98%	63.13%			74.30%				
Orange clownfish	Gsdf	63.26%	62.33%							
	Gsdf-like	57.14%	56.48%				91.08%			
Tiger tail seahorse	Gsdf	47.09%	44.91%							
	Gsdf-like	45.62%	44.24%					74.50%		
Pinecone soldierfish	Gsdf	59.13%	57.75%							
	Gsdf-like	58.13%	56.81%						97.62%	
Rainbow trout	Gsdf (Gsdf2)	48.43%	46.73%							
	Gsdf-like (Gsdf1)	40.89%	41.49%							40.70%
Asian bonytongue	Gsdf	36.15%	36.15%							
Spotted gar	Gsdf	33.49%	31.05%							

Supplementary Table 4. List of analysed genes, primers and fluorogenic probes used for real time PCR assays (qPCR)

Gene	Sequence (5' → 3') [a]	nM [d]	Amplicon size	cDNA dilution	First published
gsdf1 (JQ755271) [b]	Fw: ACAGAGCTGCCTTGCAATCC	200	99 bp	1/40 (samples from adults)	[A][18]
	Rv: TCTTGTATGACAAAGCCTGCC	200		1/10 (all the other samples)	
gsdf2 (JQ755272) [b]	Fw: TAGTAACTGATGAAATGATTGAGC	200	149 bp	1/40 (samples from adults)	[A][18]
	Rv: GTGCGAGAGAAATGCCTACAG	200		1/10 (all the other samples)	
cyp19a1a (AJ311177) [b]	Fw: TCCTCGCCGCTACTTCCA	300	65 bp	1/5	[B][63]
	Rv: TGGCGATGTGCTTACCAACA	300			
	Pr: [6 ~ FAM]CATTCGGTTCAGGCCCTCGCG[TAMRA]	100			
18S rRNA	Fw: GCATGCCGGAGTCTCGTT	900	71 bp	1/80	[B][63]
	Rv: TGCATGGCCGTTCTTAGTTG	900			
	Pr: [6 ~ FAM]TTATCGGAATTAACCAGAC[TAMRA]	200			
luc1 (MH759210.1) [c]	Fw: TACAACACCCCAACATCTTCGA	900	67 bp	1/40	[C][61]
	Rv: GGAAGTTCACCGGCGTCAT	900			
	Pr: [6 ~ FAM]CGGGCGTGGCAGGTCTTCCC[TAMRA]	200			

[a] Forward (Fw) and reverse (Rv) primers were obtained from Invitrogen™ (Life Technologies). Hydrolysis probe (Pr) was purchased from Eurofins Genomics, Germany.
[b] GenBank accession no. for sea bass (*Dicentrarchus labrax*) gene.
[c] GenBank accession no. for *Photinus pyralis* voucher KSH 11044.
[d] Amount of primer or probe in the PCR.

A. Crespo, B.; Gómez, A.; Mazón, M.J.; Carrillo, M.; Zanuy, S. Isolation and Characterization of Ff1 and Gsdf Family Genes in European Sea Bass and Identification of Early Gonadal Markers of Precocious Puberty in Males. *Gen Comp Endocrinol* **2013**, *191*, 155–167, doi:10.1016/j.ygcen.2013.06.010.

B. Rocha, A.; Zanuy, S.; Carrillo, M.; Gómez, A. Seasonal Changes in Gonadal Expression of Gonadotropin Receptors, Steroidogenic Acute Regulatory Protein and Steroidogenic Enzymes in the European Sea Bass. *Gen Comp Endocrinol* **2009**, *162*, 265–275, doi:10.1016/j.ygcen.2009.03.023.

C. Zapater, C.; Rocha, A.; Molés, G.; Mascoli, A.; Ibañez, S.; Zanuy, S.; Gómez, A. Functional Activity of Recombinant Forms of Amh and Synergistic Action with Fsh in European Sea Bass Ovary. *Int J Mol Sci* **2021**, *22*, doi:10.3390/ijms221810092.
